# MSBWO: A Multi-Strategies Improved Beluga Whale Optimization Algorithm for Feature Selection

**DOI:** 10.3390/biomimetics9090572

**Published:** 2024-09-22

**Authors:** Zhaoyong Fan, Zhenhua Xiao, Xi Li, Zhenghua Huang, Cong Zhang

**Affiliations:** 1 School of Information and Artificial Intelligence, Nanchang Institute of Science & Technology, Nanchang 330108, China; 2 School of Computer Science and Technology, Hubei Business College, Wuhan 430079, China

**Keywords:** feature selection, beluga whale optimization, binary optimizer, global optimization

## Abstract

Feature selection (FS) is a classic and challenging optimization task in most machine learning and data mining projects. Recently, researchers have attempted to develop more effective methods by using metaheuristic methods in FS. To increase population diversity and further improve the effectiveness of the beluga whale optimization (BWO) algorithm, in this paper, we propose a multi-strategies improved BWO (MSBWO), which incorporates improved circle mapping and dynamic opposition-based learning (ICMDOBL) population initialization as well as elite pool (EP), step-adaptive Lévy flight and spiral updating position (SLFSUP), and golden sine algorithm (Gold-SA) strategies. Among them, ICMDOBL contributes to increasing the diversity during the search process and reducing the risk of falling into local optima. The EP technique also enhances the algorithm′s ability to escape from local optima. The SLFSUP, which is distinguished from the original BWO, aims to increase the rigor and accuracy of the development of local spaces. Gold-SA is introduced to improve the quality of the solutions. The hybrid performance of MSBWO was evaluated comprehensively on IEEE CEC2005 test functions, including a qualitative analysis and comparisons with other conventional methods as well as state-of-the-art (SOTA) metaheuristic approaches that were introduced in 2024. The results demonstrate that MSBWO is superior to other algorithms in terms of accuracy and maintains a better balance between exploration and exploitation. Moreover, according to the proposed continuous MSBWO, the binary MSBWO variant (BMSBWO) and other binary optimizers obtained by the mapping function were evaluated on ten UCI datasets with a random forest (RF) classifier. Consequently, BMSBWO has proven very competitive in terms of classification precision and feature reduction.

## 1. Introduction

With the advent of the information age, we have witnessed an unprecedented surge in data volume across various domains, ranging from engineering [1] to ecology [2] and from information technology [3] to manufacturing [4] and management [5]. The complexity of the problems in these fields is increasing and is often characterized by multiple objectives [6] and high-dimensional characteristics [7]. The high dimensionality and redundancy inherent in raw datasets can lead to excessive consumption of computational resources, adversely affecting the efficacy of learning algorithms. Thus, selecting more important data, even in datasets with a limited amount of data, is essential for increasing classification success. FS has emerged as an essential data preprocessing technique that has garnered substantial interest over recent decades. This method increases the classification accuracy and reduces the data size by selecting the most appropriate subset of features from the original dataset [8].

FS encompasses a spectrum of methods, broadly classified into filter, wrapper, and hybrid approaches [9]. Among them, filters are faster than wrappers, but they ignore the relationships among features and cannot deal with redundant information. While wrappers are relatively computationally expensive, they can attain better results than filters because of the utilization of learning techniques in the evaluation process [10]. The quest for the best feature subset has been a foundation of FS, with the determination of this subset relying heavily on the search methodologies and evaluation strategies of the candidate features. The evolution of optimization algorithms in FS has seen a transition from traditional full search, random search, sequential search, and incremental search methods to metaheuristic search approaches [8]. Metaheuristic algorithms (MAs) have become prevalent because of their ability to navigate large search spaces efficiently and effectively, avoiding the obstacles of local optima while seeking global optima [11,12]. Various metaheuristic methods, including several recent algorithms, have been applied to address FS problems [13,14,15,16,17,18]. Moreover, there are also some hybridizations or improved optimizers in the FS techniques [19,20,21,22,23]. The reason for the appearance of many such works on FS problems is that no FS technique can address all the varieties of FS problems. Hence, we need extensive opportunities to develop new efficient models for FS cases [24].

The BWO is a recently proposed population-based metaheuristic with promising optimization capabilities for addressing continuous problems [25]. The construction of BWO is inspired mainly by the behaviours of beluga whales, including swimming, preying, and whale fall. The BWO is a derivative-free optimization technique that is easy to implement. Compared with the whale optimization algorithm (WOA) [26], the grey wolf optimizer (GWO) [27], particle swarm optimization (PSO) [28], and other algorithms have local solid development capabilities. The main merit of this optimizer is the equilibrium between exploration and exploitation that ensures global convergence. Owing to its excellent advantages, BWO, or its modified version, has been employed in many fields, such as image semantic segmentation [29], cluster routing in wireless sensor networks [30], landslide susceptibility modelling [31], speech emotion recognition [32], short-term hydrothermal scheduling [33], and demand-side management [34]. In addition, some modified versions of BWO have been developed to accomplish specific optimization problems [35,36,37]. However, as a novel optimizer, BWO has been poorly studied for its effectiveness in more problems. In other words, even though this method is an excellent optimizer, it also faces some challenges in terms of improving the search ability, accelerating the convergence rate, and addressing complex optimization problems. It is necessary to extend the application fields of BWO to make this optimizer more worthy.

Although the BWO algorithm can achieve certain optimization effects in the early stages of the algorithm, in the later stages, due to insufficient population diversity and a singular exploration angle, the algorithm often has difficulty obtaining better solutions and is prone to falling into local optima. Furthermore, as the problem′s dimensions and complexity increase, the optimization capability of the BWO algorithm decreases, exploration accuracy decreases, the convergence speed decreases, and it becomes difficult to find other high-quality solutions [35,37]. The food chain embodies the principle of survival of the fittest in nature, and each organism has certain limitations in its survival strategy [22]. These limitations inspire us to deeply analyze and improve the biological behaviour-based mathematical models when designing evolutionary algorithms that simulate biological habits. Although the existing evolutionary algorithms can address many optimization problems, by constructing mathematical models that optimize biological habits, and by refining some mathematical theories, we can construct excellent mathematical models for solving optimization problems, which has the potential to further enhance the performance of the algorithms [38].

To improve the effectiveness of the original BWO and help it overcome some physiological limitations, this paper introduces several mathematical theories. First, improved circle mapping (ICM) [39] and dynamic opposition-based learning (DOBL) [40] were introduced to increase the diversity of an algorithm during the search process, thereby reducing the risk of falling into local optima and enhancing the search efficiency and accuracy. Second, the EP of GWO [27] was integrated to maintain a subpopulation composed of the best individuals, which guided the main population to evolve towards the global optimum, enhancing the algorithm′s ability to escape from local optima. Third, we integrated the SLFSUP strategy so that the MSBWO could conduct a more detailed and in-depth search within local areas, enhancing the rigor and accuracy of the development of local spaces. Finally, by introducing the Gold-SA [41] to update the population, we accelerated the convergence speed of the algorithm while maintaining the diversity of the population and improving the quality of the solutions. We tested MSBWO on twenty-three benchmark continuous problems. Simultaneously, we interrogated the feature selection problem to evaluate this proposed approach.

The main contributions of this paper are as follows:

◆ Four improvement strategies, namely, ICMDOBL population initialization, EP, SLFSUP, and Gold-SA, were used to improve the optimization performance of the BWO algorithm.
◆ Twenty-three global optimization tasks for intelligent optimization algorithm testing were used to evaluate the proposed MSBWO and compare it with other conventional and SOTA advanced metaheuristic approaches.
◆ The developed MSBWO was transformed into a binary model for tackling FS problems for the first time. Furthermore, the binary MSBWO was compared with other FS techniques on several UCI datasets.

The structure of this article is as follows: A detailed description of the standard BWO exploration and exploitation process is presented in Section 2. Section 3 introduces MSBWO, which incorporates several strategies, and proposes MSBWO for feature selection tasks. The experimental setup and results analysis of this study are shown in Section 4. Finally, in the fifth section, the conclusions and description of the work are given. To aid in understanding, this article includes a comprehensive list of relevant abbreviations, summarized in Table 1.

## 2. Original BWO

The beluga whale, adept at navigating and hunting in aquatic environments, serves as the inspiration for the BWO algorithm. This novel biomimetic optimization approach emulates the social behaviours and hunting strategies of beluga whales to address optimization challenges. In BWO, the exploration phase is akin to the belugas′ use of echolocation to detect and track prey, representing the algorithm′s global search for optimal solutions. Conversely, the exploitation phase mirrors the focused hunting tactics employed by these whales once a target has been located, signifying the algorithm′s searching refinement in a promising area. Additionally, the whale fall phase introduces a unique mechanism that simulates the natural phenomenon of a whale falling to the ocean floor after death, which serves as an ecological disturbance that can lead to new areas of exploration and potentially escape from local optima in the search space.

### 2.1. Initialization

The locations of beluga whales are regarded as search agents. In the initialization phase, the initial population is generated randomly, and the fitness value of each individual is computed. The population initialization model is expressed as:

(1) X=rand(m,n)·(Ub−Lb)+Lb

where *Ub* and *Lb* are the upper and lower boundaries of the optimization problem to be solved, *m* is the population size of beluga whales, and *n* is the dimension of the solution. In each iteration, BWO transfers from exploration to exploitation depending on the balance factor *B_f_*, which is similar to the Harris hawks optimizer (HHO) [42]. The balance factor *B_f_* is modelled as:

(2) $$B_{f}=B_{0}\left(1-T/2T_{max}\right)$$

where $B_{0}$ is a random number between (0, 1), T is the current iteration, and $T_{max}$ is the maximum iterative number. Exploration occurs when the balance factor $B_{f}>0.5$, resulting in pairs of two beluga whales swimming closely together in a synchronized or mirrored manner. In the case of $B_{f}\leq 0.5$, the exploitation phase occurs, engaging in the preying behaviour of beluga whales.

### 2.2. Exploration Phase

The exploration phase of BWO simulates the pair swimming behaviour of beluga whales. They randomly move in a synchronized or mirrored manner, expressed as follows:

(3) $$\{\begin{array}{l} X_{i,j}^{T+1}=X_{i,p_{j}}^{T}+\left(X_{r,p_{1}}^{T}-X_{i,p_{j}}^{T}\right)\left(1+r_{1}\right)sin\left(2\pi r_{2}\right),\;\;\;j=even\\ X_{i,j}^{T+1}=X_{i,p_{j}}^{T}+\left(X_{r,p_{1}}^{T}-X_{i,p_{j}}^{T}\right)\left(1+r_{1}\right)cos\left(2\pi r_{2}\right),\;\;\;j=odd \end{array}$$

where $p_{j}\left(j=1,\;2,\cdots ,d\right)$ is a random number selected from the *d* dimension, $X_{i,p_{j}}$ is the position of the *i*th beluga whale in the $p_{j}$ dimension, $X_{r,p_{1}}$ is the current position of the *r*th beluga whale randomly selected, $r_{1}$ and $r_{2}$ are random numbers between (0, 1), and $sin\left(2\pi r_{2}\right)$ and $cos\left(2\pi r_{2}\right)$ indicate that the fins of the mirrored beluga whales are facing the surface.

### 2.3. Exploitation Phase

The exploitation phase of BWO mimics the preying behaviour of beluga whales. Beluga populations communicate with each other and share information on their positions to cooperatively forage and move according to the locations of nearby beluga whales. To enhance the algorithm′s convergence, the Lévy flight (LF) strategy is employed during the exploitation phase, which can be represented as:

(4) $$X_{i}^{T+1}=r_{3}X_{best}^{T}-r_{4}X_{i}^{T}+C_{1}\times L_{F}\cdot \left(X_{r}^{T}-X_{i}^{T}\right)$$

where $C_{1}=2r_{4}\left(1-T/T_{max}\right)$ is the random jump strength, $r_{3}$ and $r_{4}$ are random numbers between (0, 1), $X_{r}$ is the current position for a random beluga whale, and $X_{best}$ is the best position among beluga whales. $L_{F}$ is the LF function, which is calculated as follows:

(5) $$L_{F}=0.05\times \frac{u\times \sigma }{{\left|v\right|}^{1/\beta }}$$

(6) $$\sigma ={\left(\frac{\Gamma \left(1+\beta \right)\times sin\left(\pi \beta /2\right)}{\Gamma \left(\left(1+\beta \right)/2\right)\times \beta \times 2^{\left(\beta -1/2\right)}}\right)}^{1/\beta }$$

where u and v are normally distributed random numbers, β is the default constant equal to 1.5, and Γ(x) is the Γ function.

### 2.4. Whale Fall Phase

If the balance factor $B_{f}\leq W_{f}$, whale fall occurs. $W_{f}=0.1-0.05T/T_{max}$ is the probability of whale fall of individual beluga whales. The whale fall is a phenomenon in which beluga whales are threaten by killer whales and humans during migration and foraging. The dead beluga whale falls into the deep seabed. Afterwards, to maintain the population size, the updated position is established using the positions of the current beluga whale, random individual beluga whales, and the step size of the whale fall:

(7) $$X_{i}^{T+1}=r_{5}X_{i}^{T}-r_{6}X_{r}^{T}+r_{7}X_{step}$$

where $r_{5}$, $r_{6}$, and $r_{7}$ are random numbers between (0, 1), and $X_{step}$ is the step size of the whale fall established as:

(8) $$X_{step}=\left(Ub-Lb\right)e^{-C_{2}T/T_{max}}$$

where $C_{2}$ is the step factor, which is related to the probability of a whale fall and population size, $C_{2}=2W_{f}\times m$.

## 3. Proposed MSBWO

The proposed MSBWO introduces four fruitful strategies: (1) the ICMDOBL population diversity strategy, (2) the EP mechanism, (3) the SLFSUP, and (4) the Gold-SA population update mechanism. In addition, the feature selection problem can be solved by updating MSBWO to a binary version.

### 3.1. ICMDOBL Population Diversity

Owing to the random population generation of the BWO algorithm, it can lead to an uneven population distribution, which may result in reduced population diversity and lower population quality, thereby affecting the convergence of the algorithm. Chaotic mapping is characterized by uncertainty, irreducibility, and unpredictability [43], which can lead to a more uniform population distribution than probability-dependent random generation. MSBWO generates an initial population with chaotic mapping to increase the diversity of potential solutions. There are common chaotic mappings, such as logistic mapping, tent mapping [44], sine mapping [45], and circle mapping [46]. Circle mapping is more stable and has a higher coverage rate of chaotic values. Considering that circle mapping takes values more densely between [0.2, 0.6], the circle mapping formula is improved as follows:

(9) $$X_{i+1,j}=mod\left(3.85X_{i,j}+0.4-\frac{0.7}{3.85\pi }sin\left(3.85\pi X_{i,j}\right),1\right)$$

where $X_{i,j}$ represents the sequence value of the *i*th beluga whale on the *j*th dimension, and $X_{i+1,j}$ is the chaotic sequence value of the (*i*+1)th beluga whale on the *j*th dimension. Then, the values are scaled and shifted to generate $X_{Circle}$ with values between Lb and Ub for each dimension:

(10) $$X_{Circle}=\left(Ub-Lb\right)\cdot X_{i,j}+Lb$$

DOBL [40,47] is introduced to further increase population diversity and improve the quality of the initial solutions. The specific formula is expressed as follows:

(11) $$X_{DOBL}=X_{Circle}+r_{8}\times \left(r_{9}\times \left(L_{b}+U_{b}-X_{Circle}\right)-X_{Circle}\right)$$

where $X_{Circle}$ is the population established with the ICM method, as shown in Equation (10), and $r_{8}$ and $r_{9}$ are random numbers between (0, 1). The DOBL generates $X_{Circle}$ and an opposition population $X_{DOBL}$ and then merges these two populations into a new population, $X_{new}=\left\{X_{DOBL}\cup X_{Circle}\right\}$. The fitness values of $X_{new}$ are calculated, and the greedy strategy is used for full competition within the new population. The best *N* individuals are then selected as the initial population. Using ICMDOBL, MSBWO starts iterating from individuals with better fitness, thereby enhancing the convergence.

### 3.2. EP Strategy

In the case of location updating, beluga whales always use the best whale as prey. If the prey has fallen into a local optimum, all subsequent search agents will converge to it, leading to a premature convergence of the algorithm. In the GWO algorithm [27], a hierarchical system was proposed to update the positions according to the mean position of the first best three grey wolves to avoid the shortcomings caused by guiding a single search agent.

Inspired by GWO, the EP strategy is integrated into MSBWO. The first three best solutions obtained thus far, and their weighted average, are included as the candidate elites in the EP. The first three best solutions are conducive to exploration, whereas the weighted average position represents the evolutionary trend of the entire superior population, which is beneficial for exploitation. Position updating is guided by the agent randomly selected from the EP, improving the algorithm′s ability to escape from local optima.

The EP strategy is modelled as:

(12) $$\{\begin{array}{l} Elite^{T}=\left[X_{best\_1}^{T},X_{best\_2}^{T},X_{best\_3}^{T},X_{mean}^{T}\right]\\ X_{mean}^{T}=\overset{best\_num}{\underset{i=1}{\sum }}\theta_{i}X_{best\_i}^{T},\;\;\theta_{i}=\frac{\omega_{i}}{\sum_{j=1}^{best\_num}\omega_{j}}\;\\ \omega_{i}=\frac{3f_{best\_3}-f_{best\_i}}{3f_{best\_3}-f_{best\_1}} \end{array}$$

where $f_{best\_i}$ is the fitness value, and best_num is set to 3.

### 3.3. SLFSUP Strategy

In the exploitation phase, BWO uses LF with a fixed step to improve its convergence. However, at different stages of the algorithm, the expected step of LF may vary. The larger the step of LF is, the easier it is to find the optimum result, but it reduces the search precision. The smaller the step size is, the higher the search precision, but it reduces the search speed. Therefore, the step-adaptive LF strategy is used in MSBWO to improve its exploitation and convergence accuracy. In the early stages of iteration, MSBWO uses LF with a larger step so that it can fully exploit the solution space, whereas it becomes more refined in the later stage with a decreasing LF step. The step-adaptive LF strategy is calculated as follows:

(13) $$L_{F}^{\prime }=0.05\times \left(1-T/\left(T_{max}/2\right)\right)\times e^{-T/T_{max}}\cdot \frac{u\times \sigma }{{\left|v\right|}^{1/\beta }}$$

As stated in Equation (4), position updating in the exploitation of BWO involves a random agent, the best agent, and the current agent. There may still be omissions for the possible solution. In WOA [26], the spiral updating position strategy is used according to the position of the prey, namely, the best solution obtained, and the position of the whale adjusts the distance when searching for prey. Such a strategy can make full use of the regional information and improve the search capabilities. Therefore, MSBWO introduces this method to enhance the algorithm′s rigor and accuracy in the development of the local space and to strengthen the local search ability.

The position updating model in the exploitation process based on the SLFSUP is as follows:

(14) $$X_{i}^{T+1}=r_{3}\;X_{EP}^{T}-r_{4}X_{i}^{T}+C_{1}\times L_{F}^{\prime }\cdot \left(X_{r}^{T}-X_{i}^{T}\right)\cdot \left|X_{EP}^{T}-X_{i}^{T}\right|\;\times cos\left(2\pi l\right)\times e^{bl}$$

where $X_{EP}$ is the position of an agent randomly selected from $Elite^{T}$, $X_{r}$ is the current position for a random beluga whale to maintain its diversity, *b* is a constant for defining the shape of the spiral, and l is a random number in [−1, 1]. b is set to 1 in MSBWO.

### 3.4. Golden-SA Update Mechanism

Inspired by the relationship between the sine function and a one-unit radius circle, the Gold-SA [41] scans all values of the sine function. The algorithm has strong global searching capabilities. The golden section ratio is used in the position updating of Gold-SA so that it can completely scan the search space as much as possible, thus accelerating convergence and escaping from local optima.

In the MSBWO, the Gold-SA mechanism is utilized to update the beluga whale population to increase the global search ability. The position updating with Gold-SA is given as follows:

(15) $$X_{i}^{T+1}=X_{i}^{T}\left|\sin\left(r_{10}\right)\right|+r_{11}\sin\left(r_{10}\right)\left|x_{1}\times X_{EP}^{T}-x_{2}\times X_{i}^{T}\right|$$

where $r_{10}$ is a random number in the range [0, 2π] and where $r_{11}$ is a random number in the range [0, π]. $x_{1}$ and $x_{2}$ are the coefficients obtained via the golden section method, which aims at narrowing the search space and allowing the current value to approach the target value. They can be expressed as follows:

(16) $$\{\begin{array}{l} x_{1}=a\tau +b\left(1-\tau \right)\\ x_{2}=a\left(1-\tau \right)+b \end{array}\;\;$$

where $\tau =\left(\sqrt{5}-1\right)/2$ is the golden number, and the initial values of a and b are −π and π, respectively.

**Figure 1:**
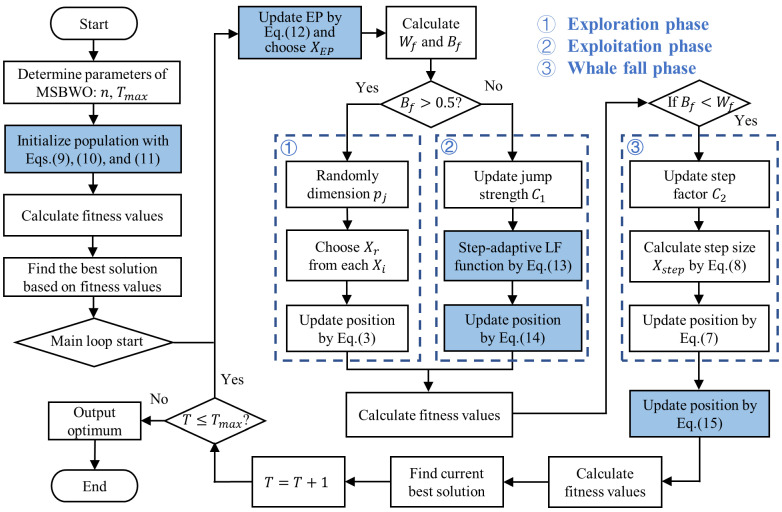
Flowchart of MSBWO; the parts that have been improved are highlighted with coloured boxes.

The proposed improvement strategies are applied to BWO, and the flow chart of the MSBWO is shown in Figure 1.

### 3.5. Binary MSBWO

FS is a binary decision optimization problem with a theoretical solution that is exponential, using 1 to represent the selection of the feature, and 0 to represent the non-selection of the feature. As an improved algorithm of the original BWO, the proposed MSBWO has a greatly improved search performance. Therefore, this study applies it to obtain a better feature subset. However, to apply MSBWO to the FS problem, the search space of the agents needs to be restricted. Moreover, binary transformation is required to map the continuous values to the corresponding binary values [48].

To address the above issues, Equations (17) and (18) are used for initialization:

(17) $$X_{i}=\left[X_{i}^{j}\right],\;1\leq i\leq m,\;1\leq j\leq d$$

(18) $$X_{i}^{j}=\{\begin{array}{c} 0,\;\;\;if\;rand<0.5\\ 1,\;\;\;if\;rand\geq 0.5 \end{array},\;\;\;1\leq i\leq m,\;1\leq j\leq d$$

where $X_{i}^{j}$ is the *j*th component of the *i*th agent, d is the size of the features, and rand is a random number between (0, 1).

After position updating, the sigmoid function is used for discretization. The transfer function and position updating equation selected in this paper are shown in Equations (19) and (20).

(19) $$S\left(X_{i}^{j}\left(t\right)\right)=\frac{1}{1+exp\left(X_{i}^{j}\left(t\right)\right)}$$

(20) $$X_{i}^{j}\left(t+1\right)=\{\begin{array}{c} 0,\;\;\;if\;rand<S\left(X_{i}^{j}\left(t+1\right)\right)\\ 1,\;\;\;if\;rand\geq S\left(X_{i}^{j}\left(t+1\right)\right) \end{array}$$

As a combination optimization, the FS has two main goals. One is to improve the classification performance, and the other is to minimize the number of selected features. Therefore, the fitness function is shown in Equation (21).

(21) $$Fitness=r_{12}E_{R}\left(D\right)+r_{13}\left|S\right|/\left|D\right|$$

where $E_{R}\left(D\right)$ represents the classification error rate of the RF classifier, *D* denotes the number of features in the original dataset, and S denotes the length of the selected feature subset. $r_{12}$ and $r_{13}$ are used to balance the relationship between the error rate and the ratio of selected features; $r_{12}\in \left[0,1\right]$, $r_{13}=1-r_{12}$.

### 3.6. Computational Complexity

To gain a better understanding of the implementation process of the MSBWO algorithm proposed in this paper, the computational complexity of MSBWO is analyzed as follows. The computational complexity of the MSBWO relies on three main steps: initialization, fitness evaluation, and updating of the beluga whale. In the initialization phase of MSBWO, the computational complexity of each agent is assumed to be O(d), where d is the dimension of a particular problem. The computational complexity of ICMDOBL is O(n×d), where n is the population size. After entering the iteration, the computational complexity of EP is O(n×logn+n). In the exploration and exploitation phases, the novel exploitation mechanism replaces the original exploration mechanism, and the computational complexity is similar to that of BWO, which is represented as $O\left(n\times d\times T_{max}\right)$. The computation of the whale fall phase can also be approximated as $O\left(0.1\times n\times T_{max}\right)$, similar to BWO. Additionally, the Gold-SA is an extra searching strategy whose computational complexity is O(n×d). Therefore, the total computational complexity of MSBWO is evaluated approximately as $O\left(n\times d+n\times T_{max}\times \left(1.1+logn+2\times d\right)\right)$. Thus, the MSBWO algorithm proposed in this paper has greater computational complexity than the original BWO algorithm.

## 4. Experiments and Results Analysis

While confirming the performance of MSBWO, sufficient targeted experiments were performed in this work. The results of the comparative observations are discussed in a comprehensive analysis. To decrease the influence of external factors, every task in this work was conducted in the same setting. With respect to the parameter settings for the metaheuristic algorithms, a total of 50 search agents were set up, except for those used for the FS experiments, and multiple iterations were completed. To reduce the impact of experimental randomness, each algorithm was executed on the benchmark function 30 times.

We applied two statistical performance measures, the mean and standard deviation (std), which represent the robustness of the tested methods, to assess the optimization ability of the MSBWO. Furthermore, some statistically significant results were used to estimate the success of the MSBWO. In this study, we utilized the Wilcoxon rank-sum test to analyze the significant differences in the statistical results among the compared approaches. The significance level was set to 0.05. In the results of the Wilcoxon rank-sum test, the rows identified by ‘+/=/−’ are the results of the significance analysis. The symbol ‘+’ indicates that MSBWO outperforms the other compared approaches significantly, ‘=’ indicates that there is no significant difference between MSBWO and the other compared approaches, and ‘−’ indicates that MSBWO is worse than the other compared methods. Additionally, the Friedman test was applied to express the average ranking performance (denoted as ARV) of all the compared approaches more closely for further statistical comparison.

Section 4.1 presents an extensive scalability analysis to perform a more comprehensive investigation into the efficiency of MSBWO on CEC2005 benchmark problems [49], as shown in Table 2, Table 3 and Table 4. Section 4.2 investigates the impact of different optimization strategies on the final search for the global optimal solution. In Section 4.3, MSBWO is compared with other conventional MAs in terms of convergence speed and accuracy on the race functions. Section 4.4 compares MSBWO to the SOTA metaheuristic approaches that were introduced in 2024. In Section 4.5, 10 datasets are selected from the UCI machine learning library to test the performance of the binary MSBWO in FS.

All the experiments were performed on a 2.60 GHz Intel i7-10750H CPU equipped with 16 GB of RAM and Windows 10 OS and were programmed in MATLAB R2023b.

### 4.1. Scalability Analysis of MSBWO

The dimensions of the optimization problems affect the efficiency of the algorithm. Therefore, it is necessary to conduct an extensive scalability analysis to perform a more comprehensive investigation into the efficiency of MSBWO. The purpose of the scalability evaluation experiment is to compare the performance of MSBWO with that of BWO as the number of dimensions increases. In this section, we test the first 13 of 23 benchmark problems with dimensions of 100, 200, 500, 1000, and 2000.

In each experiment, 30 dependent runs were applied to each method to reduce the influence of randomness on the experimental results. Additionally, the maximum number of iterations was set to 500, and the population size was set to 50. The parameter initialization of all the methods was the same as that of their original references.

The results in Table 5 present the obtained statistical values for 13 problems on each dimension. According to the statistical results in Table 5, MSBWO is more successful than BWO in addressing the optimization problems on each dimension. Despite the significant statistical results at 1000/2000 dimensions being somewhat reduced compared with the results at 100/200/500 dimensions, the solutions of each function with 1000/2000 dimensions achieved by MSBWO are much closer to the optimal solution than BWO, according to statistical measures (average values and standard deviations).

For the unimodal problems (F1-F7), MSBWO outperforms BWO, except for F5 and F6, and at 1000/2000 dimensions, which indicates that MSBWO significantly strengthens the exploitative ability of BWO at 100/200/500 dimensions. For the multimodal problems (F8-F13), the MSBWO is better than the BWO for F8 with each dimension. MSBWO shows no difference from BWO when addressing F9-F11, and both attained their optimal solutions. However, BWO is superior to MSBWO for F12 and F13 with dimensions of 1000 and 2000. That is, at 1000/2000 dimensions, the advantage of MSBWO over the original BWO is not as pronounced as it is at 100/200/500 dimensions.

From the standard deviation perspective, the standard deviations of the MSBWO on each dimension are lower than those of the BWO when solving functions F2, F4, and F7, which are equal to those of the BWO with functions F1, F3, F9, F10, and F11. This indicates that the optimization ability of MSBWO is no less than that of BWO, and this stability is not significantly affected by the number of dimensions. Although the performance of MSBWO is not superior to that of BWO on F8, MSBWO can find a satisfactory solution. Moreover, MSBWO attains a lower ‘ARV’ than BWO does in each case of dimension, which clearly reveals the superiority of MSBWO without the dependence of dimension.

It can be concluded that the strategies integrated into MSBWO facilitate the balance of exploration and exploitation and significantly enhance the search ability in different dimensions for specific problems.

### 4.2. Cross-Evaluation of the Proposed MSBWO

To verify the contributions of various improvement strategies to MSBWO, this section compares the original BWO algorithm with five incomplete versions of the MSBWO algorithm. Section 3.1, Section 3.2, Section 3.3, Section 3.4 introduce four integration strategies, including ICMDOBL, EP, SLFSUP, and Golden-SA, into the original BWO. In this section, the performance after mixing and crossing is tested and compared, mainly by means of linear combinations. In Table 6, “1” means that the mechanism was selected, and “0” means that it was not. We refer to the BWO combined with ICMDOBL as ICMDOBL_BWO, and the fusion of the BWO and EP strategies as EP_BWO. The combination of BWO and SLFSUP is denoted as SLFSUP_BWO, and the fusion of BWO and Golden-SA is denoted as GSA_BWO. In addition, the EP_GSA_BWO integrates BWO with the EP and Golden-SA strategies. The dimensions of the various methods were set to 30. Each algorithm was executed on all 23 benchmark functions 30 times.

From the horizontal comparison in Table 7, it is not difficult to find that the improvement strategies introduced into MSBWO enhance the performance of BWO to varying degrees. ICMDOBL_BWO outperforms traditional BWO on the test functions except for F12. The mechanism helps the algorithm start searching from a broader solution space, ultimately stably converging to the optimal solution. EP_BWO emphasizes the inheritance of excellent agents while maintaining population diversity, which helps the algorithm quickly converge to a high-quality area in the solution space. EP_BWO significantly outperforms BWO on the F1–F6, F12–F14, F15, and F21–F23 functions, showing good performance in maintaining population diversity and accelerating the convergence speed. SLFSUP_BWO improves the algorithm′s exploration capability in the search space by adaptively adjusting the search step size, and it significantly outperforms BWO on the F1-F4 functions. GSA_BWO updates the population position by simulating the dynamic changes in the sine waves. This strategy stands out in that it improves the algorithm′s global search capability and helps find the global optimal solution. GSA_BWO significantly outperforms BWO except for F7–F11, and F15. Each improvement strategy has unique advantages and is suitable for different types of problems. EP_GSA_BWO performs best on multimodal problems, whether multimodal or fixed-dimensional multimodal problems, as it integrates the advantages of the EP and Gold-SA strategies.

As shown by the std, EP_GSA_BWO has the best stability on most test functions. Its std is generally zero or very small, except for F8. However, GSA_BWO and EP_GSA_BWO have larger stds on F8, indicating that the Gold-SA strategy has a significant fluctuation in performance on F8. EP_BWO generally has a smaller std on the test functions, indicating good stability. The std of ICMDOBL_BWO is generally similar to that of BWO, indicating that the improvement strategy has little impact on stability.

It can be seen from the Wilcoxon signed-rank test and ARV that the variant BWO clearly enhances the performance of BWO, although each improvement strategy has unique advantages and applicable scenarios.

### 4.3. Comparison with Conventional MAs

**Figure 2:**
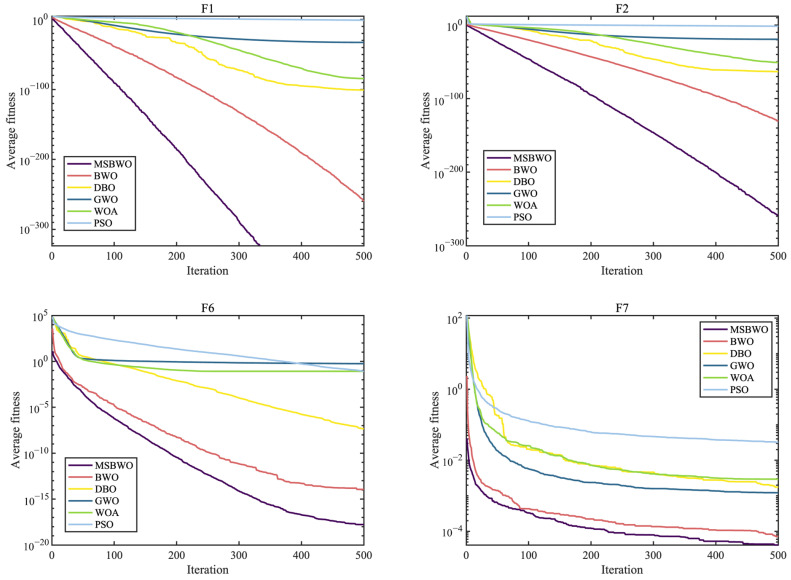
Convergence curves of MSBWO and conventional MAs on F1, F2, F6, and F7.

**Figure 3:**
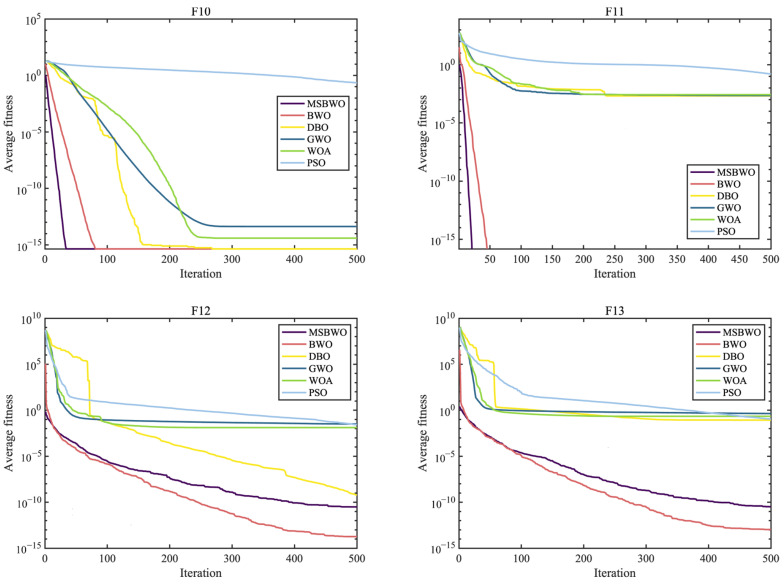
Convergence curves of MSBWO and conventional MAs on F10 to F13.

**Figure 4:**
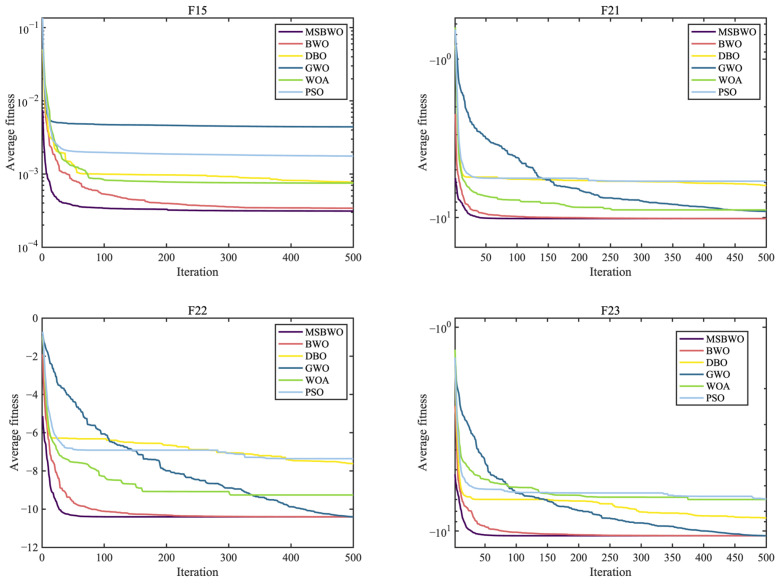
Convergence curves of MSBWO and conventional MAs on F15, F21, F22, and F23.

To further assess the optimization performance of MSBWO, in addition to BWO, we select four well-known MAs to participate in the competition, namely, dung beetle optimizer (DBO) [50], GWO [27], WOA [26], and PSO [28]. The parameter initialization of all the algorithms was the same as that of their original references. Additionally, the population size was 50, the dimension was 30, and the maximum iteration number was 500. In addition, each function was executed 30 times. Table 8 presents the statistical outcomes in terms of the mean and standard deviation (marked by ‘std’) of the proposed MSBWO compared with other selected algorithms on 23 benchmark problems. The statistical significances of values in Table 8 are shown in Table 9. Figure 2, Figure 3 and Figure 4 present the convergence curves of the three categories of algorithms.

From the statistical results listed in Table 8, MSBWO can find the best solutions, even the optimal solutions, on most of the functions, except for F12, F13, F17, and F19. For F12 and F13, the performance of MSBWO is worse than that of BWO; however, the results are close to those of BWO and are far better than those of any of the other four methods. For F17 and F19, MSBWO performs next in terms of performance to the first method, namely, DBO and PSO on F17 and PSO on F19. All algorithms present similar best average values for F16-F19 but different standard deviations.

According to the *p* values of the Wilcoxon rank-sum test, which analyzes the significant difference between the paired algorithms in Table 9, the performance of MSBWO has significantly positive differences in these four functions compared with that of the other compared methods. From the overall significant statistical results of the Wilcoxon rank-sum test on all the functions, the worst-case MSBWO produces 14 significantly better, 7 equal, and 2 significantly worse results than the PSO does, and the best case is that MSBWO overwhelmingly succeeds on almost all of the algorithms compared with GWO. It makes sense that MSBWO obtains the best ARV of 1.4565 in the Friedman test. Therefore, the conclusion can be drawn that the proposed MSBWO is the best approach with considerable advantages over five competitive swarm-based algorithms.

From the standard deviation perspective, the standard deviations of MSBWO are the lowest for 15 functions, although those of MSBWO are not the lowest for F1, F3, and F9-F11. These results indicate that the optimization ability of MSBWO is more stable than that of the other algorithms. The performance of MSBWO is not superior on F12, F13, F17, or F19; however, MSBWO can find satisfactory solutions when solving these functions.

The curves in Figure 2, Figure 3 and Figure 4 intuitively draw the convergence rates of the proposed MSBWO, BWO, DBO, GWO, WOA, and PSO for addressing the unimodal (F1, F2, F6, and F7), multimodal (F10–F13), and composition (F15, F21–F23) problems. According to Figure 2, Figure 3 and Figure 4, the MSBWO has powerful advantages in terms of the convergence rate over the other approaches in terms of F1, F2, F10, F11, F15, and F21–F23. Other approaches, especially DBO, GWO, WOA, and PSO, stagnate into local optima during early optimization on F1, F2, F6, F11, F12, and F15, whereas MSBWO has the fastest convergence rate and can obtain the best solutions on these functions. These trends indicate that the improvement in MSBWO is clearly confirmed in most cases of the unimodal, multimodal, and composition tasks.

Accordingly, these experimental results verify that the developed MSBWO has an efficient searching ability at an accelerated convergence speed, which benefits mainly from the ICMDOBL strategy and EP strategy. The ICMDOBL strategy helps the algorithm to have better initial random agents, and the individuals are more equally scattered in the global space and have a better chance to approach the global optimal solution. Moreover, the SLFSUP mechanism allows the algorithm to adjust the step size during the search process according to the current search situation, which can excellently achieve a reasonable balance between the exploitation and exploration abilities. Gold-SA also accelerates convergence and escape from local optima.

### 4.4. Comparison with SOTA Algorithms

**Figure 5:**
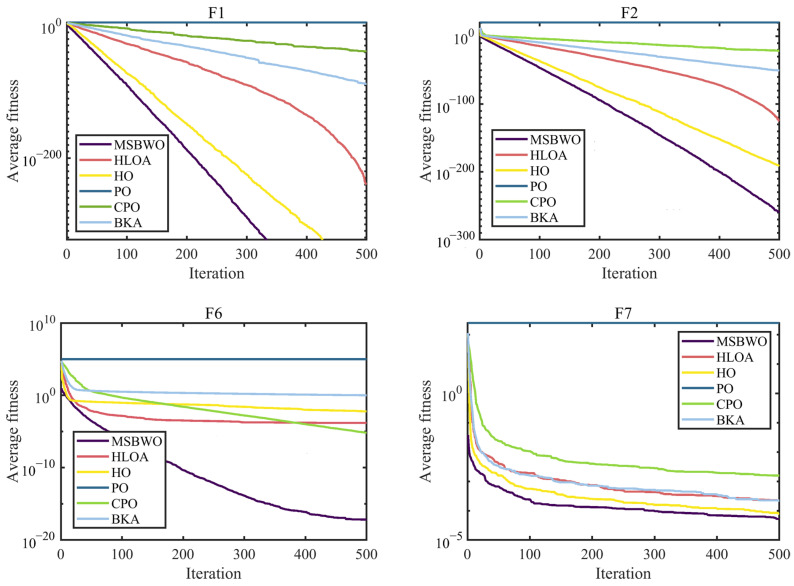
Convergence curves of the MSBWO and SOTA algorithms on F1, F2, F6, and F7.

**Figure 6:**
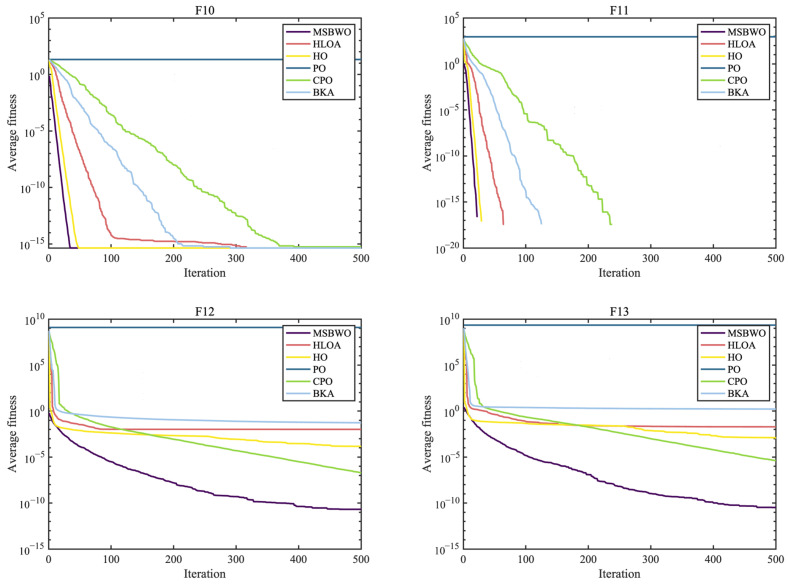
Convergence curves of the MSBWO and SOTA algorithms on F10 to F13.

**Figure 7:**
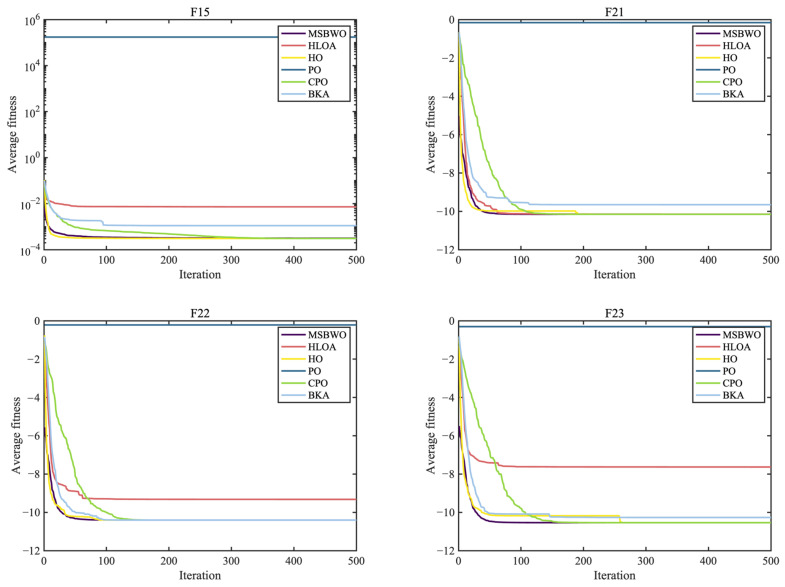
Convergence curves of the MSBWO and SOTA algorithms on F15, F21, F22, and F23.

To further investigate the advantages of the proposed MSBWO, the algorithm was compared against five SOTA MAs that were introduced in 2024, namely, the horned lizard optimization algorithm (HLOA) [51], hippopotamus optimization (HO) [52], parrot optimizer (PO) [53], crested porcupine optimizer (CPO) [54], and black-winged kite algorithm (BKA) [55]. The simulation results, including the Wilcoxon test and the Friedman test results, can be seen in Table 10. Table 11 records the *p* values of the Wilcoxon test, which were used to investigate the significant differences between MSBWO and one of the compared algorithms. The statistical significances of values in Table 10 are shown in Table 11. These results are clearly illustrated in the convergence curves in Figure 5, Figure 6 and Figure 7.

As reported in Table 10, MSBWO achieves the best solutions for approximately 78% of the functions except for F7, F14, F15, and F20. For F7, MSBWO is only worse than PO. The performance of MSBWO on F14 is worse than that of HO, CPO, and BKA. For F15, the means of HO, PO, and CPO are better than those of MSBWO. Nevertheless, according to the *p* values in Table 11, for F15, there is no significant difference between MSBWO and the PO. For F20, the solution obtained via MSBWO approaches the best solutions.

According to the ARV, the established MSBWO is ranked the best, with a value of 2.2174. Additionally, from Table 11, we observe that MSBWO significantly outperforms the other competitors in general on F1–F13, except for F9–F11, for which all the algorithms achieve the best solutions. This indicates that MSBWO is significantly better than the other five algorithms in optimizing both unimodal and multimodal problems, which reflects its excellent exploitation ability and explorative ability. For F16–F23, MSBWO shows competitive optimization capability. This shows that the performance of MSBWO in handling composition problems is not worse than those of the above advanced methods. According to the above investigations, the performance of MSBWO is superior to that of these outstanding optimizers from an overall perspective.

The convergence curves again prove the merits of MSBWO in an obvious way. From Figure 5 for unimodal functions (F1, F3, F6, and F7) and Figure 6 for multimodal functions (F10–F13), MSBWO significantly achieves the best outcome and fastest convergence rate. In contrast, other competitors, including HO, stagnate into local optima during the early stage on F6, F12, and F13. For the fixed-dimensional functions in Figure 7, although MSBWO does not achieve the fastest convergence rate, the difference in convergence speed compared with the comparative algorithms is not significant, and it also obtains the global optimal solution.

Therefore, the multiple strategies integrated into the MSBWO contribute to strengthening the balance between diversification and intensification. MSBWO effectively has a faster convergence rate or better search ability than outstanding advanced optimizers such as the HLOA, HO, PO, CPO, and BKA.

### 4.5. Feature Selection Experiment

This section presents a more comprehensive study on the proposed MSBWO in a binary manner according to the feature selection (FS) rules. Distinct test datasets were used to test the proposed approach for FS. They are available from the UCI repository, which can be obtained from the website https://archive.ics.uci.edu/datasets (accessed on 5 May 2024). The details of the datasets used for feature selection are shown in Table 12. As revealed in Table 12, the datasets contain different sizes of features and instances and belong to different subject areas. The difference in the dataset is beneficial for testing the proposed method from different viewpoints.

In this study, we chose the common RF classifier. Simultaneously, four other FS approaches, including binary GWO (BGWO), binary WOA (BWOA), binary DBO (BDBO), and binary BWO (BBWO), are regarded as competitors against the proposed BMSBWO to confirm its efficiency. In the fitness function, $r_{14}$ is set to 0.9. The number of decision trees in the RF classifier is set to 20. Additionally, the population size is 20, and the maximum number of iterations is 50. In addition, each function was executed 30 times.

The numerical results of comparing BMSBWO with BGWO, BWOA, BDBO, and BBWO on each dataset for FS problems are recorded in Table 13, Table 14, Table 15 and Table 16 in terms of fitness, error rate, mean feature selection size, and average running time. The metric mean feature selection size determines the FS ratio by dividing the FS size by the total size of the features in the original dataset. 

As outlined in Table 13, the excellent performance of the BMSBWO is evidently superior to that of the BGWO, BWOA, BDBO, and BBWO on high-dimensional samples S5-S10 in terms of fitness. For the S1-S4 datasets, the BMSBWO is not the sole best, but it still demonstrates good performance. Notably, BBWO exhibits equally excellent fitness values on the S1-S4 datasets as BMSBWO. From the final ARV obtained, the average fitness values obtained by the BMSBWO are much lower than those of the other peers. This shows that the performance of the BMSBWO is superior to those of the other algorithms. According to the final rank values in Table 14, the classification accuracy obtained via the BMSBWO still exceeds those of the other algorithms. Except for S1, S4, and S8, the classification error rates of BMSBWO are lower than those of its rivals.

The ultimate goal of feature selection is to improve the prediction accuracy and reduce the dimensionality of the prediction results. Obtaining the optimal feature subset by eliminating features with little or no predictive information and strongly correlated redundant features is the core of this work. Table 15 shows that the BMSBWO algorithm obtains a subset of features with minimum dimensionality on each dataset, indicating that the BMSBWO algorithm has a better feature selection capability. Combined with the classification error rate in Table 14, it can always filter out fewer features with a low error rate. Furthermore, BMSBWO even achieves a 0% error rate by selecting the fewest features on S4.

A comparison of the time consumption results in Table 16 reveals that BMSBWO ranks fifth, which shows that it takes more time than most binary optimizers. This is because the improved strategies, such as EP, SLFSUP, and Golden-SA, somewhat affect the time cost, which can also be seen from the computational complexity of MSBWO. Although BMSBWO has a greater time cost, considering the comprehensive performance of Table 13, Table 14 and Table 15 is worthwhile. BMSBWO outperforms the other four binary optimizers in handling the feature selection problem. Of course, how to reduce the consumption of the BMSBWO computing time while ensuring performance is still the direction of our future research.

**Figure 8:**
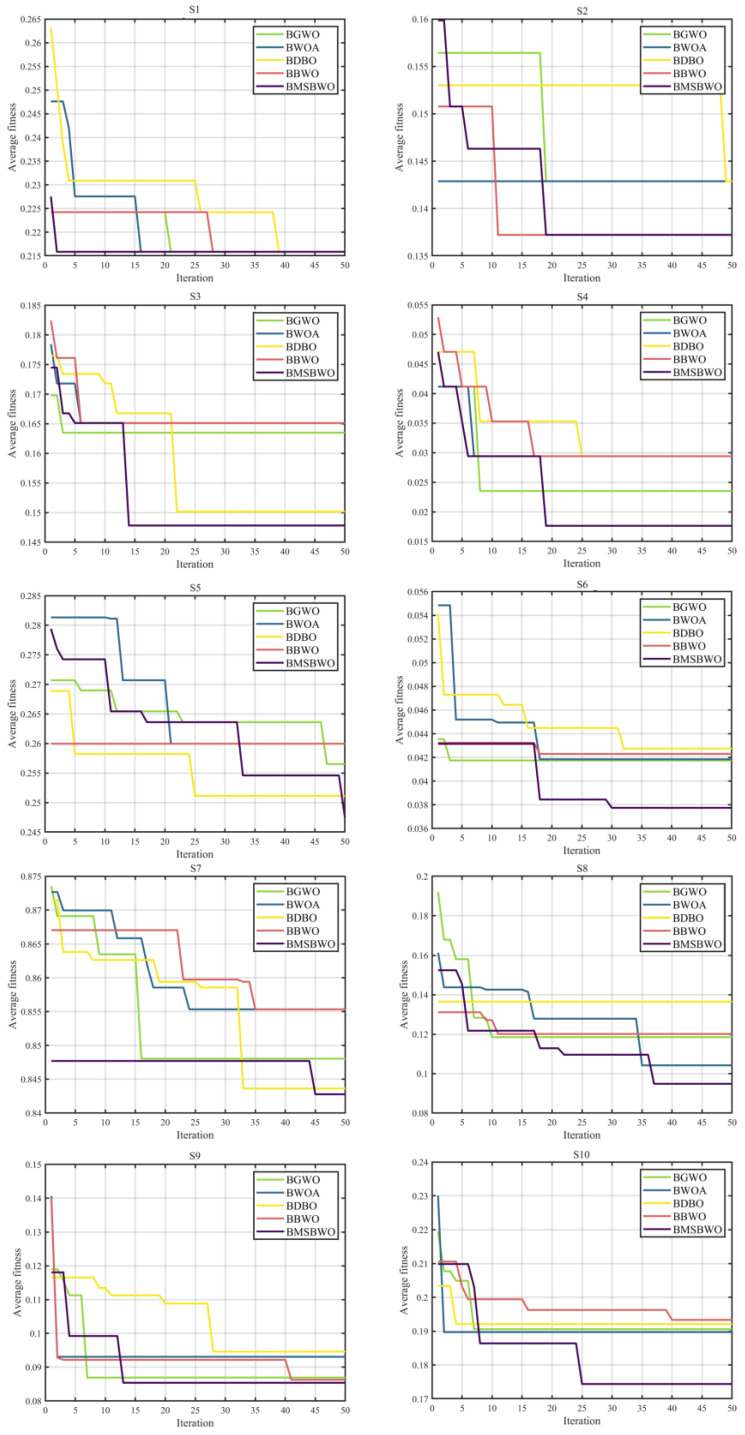
Convergence curves of the BMSBWO and other binary algorithms on 10 datasets.

The best fitness values during the iterative process are presented below as convergence curves to make the experimental results more intuitive and clearer. Figure 8 shows the convergence curves of the algorithm when comparing 10 datasets. The Y-axis shows the average fitness value under ten independent executions, and the X-axis indicates the number of iterations. The convergence values of the BMSBWO are much smaller than those of the other algorithms on approximately 80% of the benchmark datasets. It can also be observed that the MSBWO method is not prone to falling into local optima, demonstrating stronger exploration capabilities on the S5 dataset. All of these benefit from the variety of update methods provided by the ICMDOBL, EP, and SLFSUP strategies, which ensure the diversity of the population and enable the algorithm to have more opportunities to explore optimal regions.

Handling the balance between the global exploration and local exploitation search phases is a significant factor that makes BMSBWO superior to the other algorithms. The experimental results indicate that its powerful search capability enables the BMSBWO to perform excellently on a wide range of complex problems.

## 5. Conclusions and Future Works

In this paper, a novel improved BWO was constructed to optimize the diversity of population positions and the exploration–exploitation imbalance of the original BWO. The proposed optimizer is called MSBWO, which contains an initialization stage and an updating stage. In the updating stage, the EP, SLFSUP, and Golden-SA strategies were integrated with BWO to improve the rigor and accuracy of the algorithm in local space exploration, enhancing local search capabilities and accelerating the convergence speed of the algorithm.

The algorithm was applied to CEC2005 global optimization problems. The global optimization performance of MSBWO was verified by comparing it to other conventional algorithms, DBO, GWO, WOA, and PSO, as well as the SOTA algorithms HLOA, HO, PO, CPO, and BKA. The comprehensive results of the experiment indicated that the established MSBWO has excellent exploration abilities, which helps the algorithm jump out of local optimal values and accurately explore more promising regions in most cases. Thus, it is better than other optimizers in terms of search ability and convergence speed when tackling global optimization problems.

In addition, we mapped MSBWO into binary space via a mapping function based on the continuous version of MSBWO as a feature selection technique. Ten UCI datasets of different dimensions were utilized to benchmark the performance of binary MSBWO in feature selection. The experimental results clearly verified that the BMSBWO outperforms the other investigated methods with respect to fitness, mean feature selection size, and error rate measures compared with the other algorithms. This has important implications in terms of reducing the data dimensionality and improving the computing performance.

Accordingly, we can regard the proposed MSBWO algorithm as a potential global optimization method as well as a promising feature selection technique. However, the integration of improvement strategies, which contribute to enhancing the performance of the original BWO, resulted in more time costs to attain high-quality best solutions. Therefore, it is necessary to harmonize efficiency with accuracy when tackling practical problems. In future studies, a promising direction is to use the proposed method in multi-objective optimization tasks. We can also expand the application of this method to more real-life problems such as machine learning, medical applications, financial fields, and engineering optimization tasks. Moreover, research on integrating the novel BWO algorithm with other strategies to build a much better optimizer is a worthwhile endeavour.

## Figures and Tables

**Table 1:** A comprehensive list of abbreviations utilized in this article.

Abbreviations	Description
FS	Feature selection
BWO	Beluga whale optimization
MSBWO	Multi-strategies improved beluga whale optimization
ICMDOBL	Improved circle mapping and dynamic opposition-based learning
EP	Elite pool
SLFSUP	Step-adaptive Lévy flight and spiral updating position
Gold-SA	Golden sine algorithm
SOTA	State-of-the-art
BMSBWO	Binary multi-strategies improved beluga whale optimization
RF	Random forest
MAs	Metaheuristic algorithms
WOA	Whale optimization algorithm
GWO	Grey wolf optimizer
PSO	Particle swarm optimization
ICM	Improved circle mapping
DOBL	Dynamic opposition-based learning
HHO	Harris hawks optimizer
LF	Lévy flight
std	Standard deviation
DBO	Dung beetle optimizer
HLOA	Horned lizard optimization algorithm
HO	Hippopotamus optimization
PO	Parrot optimizer
CPO	Crested porcupine optimizer
BKA	Black-winged kite algorithm
BGWO	Binary grey wolf optimizer
BWOA	Binary whale optimization algorithm
BDBO	Binary dung beetle optimizer
BBWO	Binary beluga whale optimization

**Table 2:** Unimodal benchmark functions.

Function	Range	*f_min_*
$F_{1}\left(x\right)=\overset{n}{\underset{i=1}{\sum }}x_{i}^{2}$	[−100, 100]	0
$F_{2}\left(x\right)=\overset{n}{\underset{i=1}{\sum }}\left|x_{i}\right|+\overset{n}{\underset{i=1}{\prod }}\left|x_{i}\right|$	[−10, 10]	0
$F_{3}\left(x\right)=\overset{n}{\underset{i=1}{\sum }}{\left(\overset{i}{\underset{j=1}{\sum }}x_{j}\right)}^{2}$	[−100, 100]	0
$F_{4}\left(x\right)=\underset{i}{\max}\left\{\left|x_{i}\right|,1\leq i\leq n\right\}$	[−100, 100]	0
$F_{5}\left(x\right)=\overset{n-1}{\underset{i=1}{\sum }}\left[100{\left(x_{i+1}-x_{i}^{2}\right)}^{2}+{\left(x_{i}-1\right)}^{2}\right]$	[−30, 30]	0
$F_{6}\left(x\right)=\overset{n}{\underset{i=1}{\sum }}{\left(\left[x_{i}+0.5\right]\right)}^{2}\;$	[−100, 100]	0
$F_{7}\left(x\right)=\overset{n}{\underset{i=1}{\sum }}ix_{i}^{4}+random[0,1)$	[−128, 128]	0

**Table 3:** Multimodal benchmark functions.

Function	Range	$f_{min}$
$F_{8}\left(x\right)=\overset{n}{\underset{i=1}{\sum }}-x_{i}sin\left(\sqrt{\left|x_{i}\right|}\right)$	${\left[-500,\;500\right]}^{n}$ *	−418.9829×n
$F_{9}\left(x\right)=\overset{n}{\underset{i=1}{\sum }}\left[x_{i}^{2}-10cos\left(2\pi x_{i}\right)+10\right]$	${\left[-5.12,\;5.12\right]}^{n}$	0
$F_{10}\left(x\right)=-20exp\left(-0.2\sqrt{\frac{1}{n}\overset{n}{\underset{i=1}{\sum }}x_{i}^{2}}\right)-exp\left(\frac{1}{n}\overset{n}{\underset{i=1}{\sum }}cos\left(2\pi x_{i}\right)\right)+20+e$	${\left[-32,\;32\right]}^{n}$	0
$F_{11}\left(x\right)=\frac{1}{4000}\overset{n}{\underset{i=1}{\sum }}x_{i}^{2}-\overset{n}{\underset{i=1}{\prod }}cos\left(\frac{x_{i}}{\sqrt{i}}\right)+1$	${\left[-600,\;600\right]}^{n}$	0
$F_{12}\left(x\right)=\frac{\pi }{n}\left\{10sin\left(\pi y_{1}\right)+\overset{n-1}{\underset{i=1}{\sum }}{\left(y_{i}-1\right)}^{2}\left[1+10sin^{2}\left(\pi y_{i+1}\right)\right]+{\left(y_{n}-1\right)}^{2}\right\}+\overset{n}{\underset{i=1}{\sum }}u\left(x_{i},\;10,\;100,\;4\right)$ $y_{i}=1+\frac{x_{i}+1}{4}$ $u\left(x_{i},a,k,m\right)=\{\begin{array}{l} k{\left(x_{i}-a\right)}^{m}\\ 0\\ k{\left(-x_{i}-a\right)}^{m} \end{array}\begin{array}{l} x_{i}>a\\ -a<x_{i}<a\\ x_{i}<-a \end{array}$	${\left[-50,\;50\right]}^{n}$	0
$F_{13}\left(x\right)=0.1\left\{sin^{2}\left(3\pi x_{1}\right)+\overset{n}{\underset{i=1}{\sum }}{\left(x_{i}-1\right)}^{2}\left[1+sin^{2}\left(3\pi x_{1}+1\right)\right]+{\left(x_{n}-1\right)}^{2}\left[1+sin^{2}\left(2\pi x_{n}\right)\right]\right\}+\overset{n}{\underset{i=1}{\sum }}u\left(x_{i},\;5,\;100,\;4\right)$	${\left[-50,\;50\right]}^{n}$	0

* The *n* is the dimension of the solution.

**Table 4:** Fixed-dimensional multimodal benchmark functions.

Function	Range	$f_{min}$
$F_{14}\left(x\right)={\left(\frac{1}{500}+\overset{25}{\underset{j=1}{\sum }}\frac{1}{j+\sum_{i=1}^{2}{\left(x_{i}-a_{ij}\right)}^{6}}\right)}^{-1}$	${\left[-65.536,\;65.536\right]}^{2}$	1
$F_{15}\left(x\right)=\overset{11}{\underset{i=1}{\sum }}{\left[a_{i}-\frac{x_{i}\left(b_{i}^{2}-b_{i}x_{2}\right)}{b_{i}^{2}+b_{i}x_{3}+x_{4}}\right]}^{2}$	${\left[-5,\;5\right]}^{4}$	0.00030
$F_{16}\left(x\right)=4x_{1}^{2}-2.1x_{1}^{4}+\frac{1}{3}x_{1}^{6}+x_{1}x_{2}-4x_{2}^{2}+4x_{2}^{4}$	${\left[-5,\;5\right]}^{2}$	−1.0316
$F_{17}\left(x\right)={\left(x_{2}-\frac{5.1}{4\pi^{2}}x_{1}^{2}+\frac{5}{\pi }x_{1}-6\right)}^{2}+10\left(1-\frac{1}{8\pi }\right)cosx_{1}+10$	${\left[-5,\;5\right]}^{2}$	0.398
$F_{18}\left(x\right)=\left[1+{\left(x_{1}+x_{2}+1\right)}^{2}\left(19-14x_{1}+3x_{1}^{2}-14x_{2}+6x_{1}x_{2}+3x_{2}^{2}\right)\right]\times \left[30+{\left(2x_{1}-3x_{2}\right)}^{2}\left(18-32x_{1}+12x_{1}^{2}+48x_{2}-36x_{1}x_{2}+27x_{2}^{2}\right)\right]$	${\left[-2,\;2\right]}^{2}$	3
$F_{19}\left(x\right)=-\overset{4}{\underset{i=1}{\sum }}c_{i}exp\left(-\overset{3}{\underset{j=1}{\sum }}a_{ij}{\left(x_{j}-p_{ij}\right)}^{2}\right)$	${\left[0,\;1\right]}^{3}$	−3.86
$F_{20}\left(x\right)=-\overset{4}{\underset{i=1}{\sum }}c_{i}exp\left(-\overset{6}{\underset{j=1}{\sum }}a_{ij}{\left(x_{j}-p_{ij}\right)}^{2}\right)$	${\left[0,\;1\right]}^{6}$	−3.32
$F_{21}\left(x\right)=-\overset{5}{\underset{i=1}{\sum }}{\left[\left(X-a_{i}\right){\left(X-a_{i}\right)}^{T}+c_{i}\right]}^{-1}$	${\left[0,\;10\right]}^{4}$	−10.1532
$F_{22}\left(x\right)=-\overset{7}{\underset{i=1}{\sum }}{\left[\left(X-a_{i}\right){\left(X-a_{i}\right)}^{T}+c_{i}\right]}^{-1}$	${\left[0,\;10\right]}^{4}$	−10.4028
$F_{23}\left(x\right)=-\overset{10}{\underset{i=1}{\sum }}{\left[\left(X-a_{i}\right){\left(X-a_{i}\right)}^{T}+c_{i}\right]}^{-1}$	${\left[0,\;10\right]}^{4}$	−10.5363

**Table 5:** Overall results of the scalability test on 13 problems with five dimensions.

Fun	Metric	100		200		500		1000		2000	
		MSBWO	BWO	MSBWO	BWO	MSBWO	BWO	MSBWO	BWO	MSBWO	BWO
F1	Mean	0	3.1325 × 10^−240^	0	3.9284 × 10^−242^	0	2.6941 × 10^−243^	0	4.8315 × 10^−250^	0	3.3198 × 10^−247^
	std	0	0	0	0	0	0	0	0	0	0
F2	Mean	4.9784 × 10^−258^	1.7522 × 10^−122^	4.4907 × 10^−257^	1.9363 × 10^−122^	1.4864 × 10^−258^	2.0048 × 10^−122^	1.4654 × 10^−258^	3.6978 × 10^−128^	7.5977 × 10^−256^	4.7685 × 10^−127^
	std	0	7.4403 × 10^−122^	0	6.7326 × 10^−122^	0	8.5800 × 10^−122^	0	1.2181 × 10^−127^	0	1.1725 × 10^−126^
F3	Mean	0	2.8571 × 10^−236^	0	3.2202 × 10^−232^	0	2.0805 × 10^−237^	0	3.3291 × 10^−230^	0	1.1124 × 10^−228^
	std	0	0	0	0	0	0	0	0	0	0
F4	Mean	3.5853 × 10^−256^	8.0589 × 10^−121^	3.0137 × 10^−257^	2.1383 × 10^−119^	4.6305 × 10^−258^	3.7698 × 10^−121^	2.3963 × 10^−251^	2.6188 × 10^−111^	7.1375 × 10^−254^	5.4145 × 10^−105^
	std	0	4.054 × 10^−120^	0	1.1281 × 10^−118^	0	1.0441 × 10^−120^	0	6.198 × 10^−111^	0	1.9742 × 10^−104^
F5	Mean	3.4833 × 10^−3^	3.4724 × 10^−2^	2.9821 × 10^−3^	3.4543 × 10^−2^	3.3810 × 10^−3^	4.5225 × 10^−2^	2.6676 × 10^−4^	2.0253 × 10^−5^	1.6484 × 10^−4^	6.676 × 10^−5^
	std	2.9867 × 10^−3^	2.8795 × 10^−2^	2.5249 × 10^−3^	2.5515 × 10^−2^	3.4903 × 10^−3^	3.0284 × 10^−2^	7.8311 × 10^−4^	3.9018 × 10^−5^	2.9464 × 10^−4^	1.3279 × 10^−4^
F6	Mean	8.8239 × 10^−6^	3.8552 × 10^−4^	1.097 × 10^−5^	3.9011 × 10^−4^	8.1703 × 10^−6^	3.8265 × 10^−4^	2.3526 × 10^−7^	9.8573 × 10^−13^	1.2257 × 10^−6^	2.4559 × 10^−12^
	std	8.1335 × 10^−6^	1.9332 × 10^−4^	6.3314 × 10^−6^	1.8887 × 10^−4^	4.6765 × 10^−6^	1.7915 × 10^−4^	3.3537 × 10^−7^	1.2323 × 10^−12^	2.8654 × 10^−6^	4.625 × 10^−12^
F7	Mean	3.2832 × 10^−5^	5.7773 × 10^−5^	3.511 × 10^−5^	1.0244 × 10^−4^	3.717 × 10^−5^	7.8434 × 10^−5^	4.6477 × 10^−5^	7.7971 × 10^−5^	3.9363 × 10^−5^	8.8667 × 10^−5^
	std	2.8017 × 10^−5^	6.0136 × 10^−5^	2.6436 × 10^−5^	9.0007 × 10^−5^	2.8646 × 10^−5^	6.7439 × 10^−5^	3.8874 × 10^−5^	5.4964 × 10^−5^	2.0208 × 10^−5^	7.2232 × 10^−5^
F8	Mean	−1.5067 × 10^114^	−4.0675 × 10^3^	−1.3525 × 10^114^	−4.0179 × 10^3^	−2.3274 × 10^112^	−4.0622 × 10^3^	−1.9952 × 10^110^	−4.1898 × 10^5^	−4.3998 × 10^113^	−8.3797 × 10^5^
	std	6.2796 × 10^114^	1.6335 × 10^2^	5.3718 × 10^114^	1.962 × 10^2^	8.5034 × 10^112^	1.8575 × 10^2^	7.4347 × 10^110^	9.0956 × 10^−8^	2.4097 × 10^114^	1.7306 × 10^−7^
F9	Mean	0	0	0	0	0	0	0	0	0	0
	std	0	0	0	0	0	0	0	0	0	0
F10	Mean	4.4409 × 10^−16^	4.4409 × 10^−16^	4.4409 × 10^−16^	4.4409 × 10^−16^	4.4409 × 10^−16^	4.4409 × 10^−16^	4.4409 × 10^−16^	4.4409 × 10^−16^	4.4409 × 10^−16^	4.4409 × 10^−16^
	std	0	0	0	0	0	0	0	0	0	0
F11	Mean	0	0	0	0	0	0	0	0	0	0
	std	0	0	0	0	0	0	0	0	0	0
F12	Mean	2.9151 × 10^−6^	8.7024 × 10^−5^	2.7176 × 10^−6^	1.0203 × 10^−4^	2.9509 × 10^−6^	7.8079 × 10^−5^	9.5097 × 10^−10^	3.5524 × 10^−16^	1.0891 × 10^−9^	6.5309 × 10^−16^
	std	1.6047 × 10^−6^	4.9517 × 10^−5^	1.9696 × 10^−6^	5.8337 × 10^−5^	1.8946 × 10^−6^	3.8323 × 10^−5^	2.1414 × 10^−9^	3.952 × 10^−16^	1.9733 × 10^−9^	1.1161 × 10^−15^
F13	Mean	9.7534 × 10^−6^	1.1854 × 10^−4^	9.5108 × 10^−6^	1.0988 × 10^−4^	7.6788 × 10^−6^	9.9716 × 10^−5^	7.652 × 10^−7^	3.1858 × 10^−13^	1.3665 × 10^−7^	5.3749 × 10^−13^
	std	1.1971 × 10^−5^	7.7015 × 10^−5^	9.3821 × 10^−6^	9.4893 × 10^−5^	6.4942 × 10^−6^	6.0157 × 10^−5^	2.9231 × 10^−6^	7.6574 × 10^−13^	3.5755 × 10^−7^	1.2843 × 10^−12^
+/=/−		−	10/3/0	−	10/3/0	−	10/3/0	−	6/3/4	−	6/4/3
ARV		1.1154	1.8846	1.1154	1.8846	1.1154	1.8846	1.4231	1.4769	1.3846	1.6154
Rank		1	2	1	2	1	2	1	2	1	2

**Table 6:** BWO with one or more improvement strategies.

	ICMDOBL	EP	SLFSUP	Golden-SA
BWO	0	0	0	0
ICMDOBL_BWO	1	0	0	0
EP_BWO	0	1	0	0
SLFSUP_BWO	0	0	1	0
GSA_BWO	0	0	0	1
EP_GSA_BWO	0	1	0	1

**Table 7:** Results of variant BWO with the Wilcoxon signed rank test.

Fun	Metric	BWO	ICMDOBL_BWO	EP_BWO	SLFSUP_BWO	GSA_BWO	EP_GSA_BWO
F1	Mean	7.0013 × 10^−260^	1.4336 × 10^−262^	4.9908 × 10^−269^	0	0	0
	std	0	0	0	00	0	0
F2	Mean	2.8825 × 10^−133^	2.5311 × 10^−133^	3.7554 × 10^−137^	2.5676 × 10^−211^	2.4955 × 10^−227^	2.917 × 10^−224^
	std	1.0065 × 10^−132^	7.4073 × 10^−133^	1.039 × 10^−136^	0	0	0
F3	Mean	1.4033 × 10^−246^	1.0023 × 10^−245^	3.3437 × 10^−250^	0	5.9322 × 10^−305^	1.1606 × 10^−304^
	std	0	0	0	0	0	0
F4	Mean	6.2102 × 10^−128^	1.6457 × 10^−128^	2.0551 × 10^−133^	1.2706 × 10^−203^	1.0216 × 10^−217^	1.0178 × 10^−216^
	std	3.1092 × 10^−127^	5.8050 × 10^−128^	4.2469 × 10^−133^	0	0	0
F5	Mean	2.4149 × 10^−7^	1.4948 × 10^−7^	9.0321 × 10^−13^	2.9282 × 10^−7^	2.3391 × 10^−8^	8.8411 × 10^−14^
	std	3.8877 × 10^−7^	2.603 × 10^−7^	4.1987 × 10^−12^	4.438 × 10^−7^	8.7891 × 10^−8^	4.7885 × 10^−13^
F6	Mean	5.7644 × 10^−15^	9.0922 × 10^−15^	0	2.045 × 10^−14^	9.65231 × 10^−16^	0
	std	7.0405 × 10^−15^	1.6401 × 10^−14^	0	2.5569 × 10^−14^	1.2376 × 10^−15^	0
F7	Mean	7.6282 × 10^−5^	7.3459 × 10^−5^	8.1907 × 10^−5^	6.4404 × 10^−5^	4.0187 × 10^−5^	3.7322 × 10^−5^
	std	6.5538 × 10^−5^	5.7310 × 10^−5^	8.9211 × 10^−5^	5.9111 × 10^−5^	3.3717 × 10^−5^	2.7464 × 10^−5^
F8	Mean	−1.257 × 10^4^	−1.257 × 10^4^	−1.257 × 10^4^	−1.257 × 10^4^	−3.4676 × 10^148^	−1.485 × 10^117^
	std	2.8059 × 10^−9^	2.743 × 10^−9^	1.8501 × 10^−12^	3.4371 × 10^−7^	1.8709 × 10^149^	8.1337 × 10^117^
F9	Mean	0	0	0	0	0	0
	std	0	0	0	0	0	0
F10	Mean	4.4409 × 10^−16^	4.4409 × 10^−16^	4.4409 × 10^−16^	4.4409 × 10^−16^	4.4409 × 10^−16^	4.4409 × 10^−16^
	std	0	0	0	0	0	0
F11	Mean	0	0	0	0	0	0
	std	0	0	0	0	0	0
F12	Mean	1.9893 × 10^−14^	1.9976 × 10^−14^	2.7406 × 10^−32^	2.7215 × 10^−14^	4.8608 × 10^−15^	7.148 × 10^−32^
	std	2.8979 × 10^−14^	2.9732 × 10^−14^	2.3483 × 10^−32^	4.5395 × 10^−14^	5.2525 × 10^−15^	2.3217 × 10^−31^
F13	Mean	1.3464 × 10^−13^	1.5827 × 10^−13^	1.2251 × 10^−30^	2.456 × 10^−13^	2.998 × 10^−14^	6.7572 × 10^−32^
	std	2.4346 × 10^−13^	3.2734 × 10^−13^	5.9353 × 10^−30^	3.4683 × 10^−13^	5.198 × 10^−14^	1.1523 × 10^−31^
F14	Mean	0.998	0.998	0.998	0.998	0.998	0.998
	std	7.6912 × 10^−7^	2.0118 × 10^−10^	1.7869 × 10^−14^	1.7285 × 10^−9^	1.7885 × 10^−11^	2.3142 × 10^−16^
F15	Mean	3.4383 × 10^−4^	3.1749 × 10^−4^	3.4516 × 10^−4^	3.4657 × 10^−4^	3.3169 × 10^−4^	3.4711 × 10^−4^
	std	4.1472 × 10^−5^	6.4871 × 10^−6^	4.8746 × 10^−5^	6.3006 × 10^−5^	2.8705 × 10^−5^	4.6637 × 10^−5^
F16	Mean	−1.0315	−1.0316	−1.0316	−1.0315	−1.0316	−1.0316
	std	1.3142 × 10^−4^	7.4459 × 10^−5^	4.148 × 10^−5^	1.8771 × 10^−4^	2.4755 × 10^−5^	1.8592 × 10^−6^
F17	Mean	0.3988 × 10^−1^	0.3997	0.4008	0.3995	0.3981	0.3982
	std	1.3235 × 10^−3^	1.8371 × 10^−3^	4.4741 × 10^−3^	2.1497 × 10^−3^	1.8057 × 10^−4^	3.4807 × 10^−4^
F18	Mean	3.5687	3.399	3.9105	3.4292	3.0003	3.0004
	std	0.4929	0.3592	1.0680	0.4257	4.4043 × 10^−4^	4.2285 × 10^−4^
F19	Mean	−3.8594	−3.8586	−3.8543	−3.8595	−3.8622	−3.8616
	std	2.71 × 10^−3^	2.7328 × 10^−3^	4.4753 × 10^−3^	2.4504 × 10^−3^	3.6126 × 10^−4^	7.9765 × 10^−4^
F20	Mean	−3.288	−3.3023	−3.2875	−3.3004	−3.3064	−3.3185
	std	3.8818 × 10^−2^	9.5708 × 10^−3^	3.0113 × 10^−2^	2.3301 × 10^−2^	2.6131 × 10^−2^	4.4554 × 10^−3^
F21	Mean	−10.149	−10.150	−10.153	−10.147	−10.153	−10.153
	std	5.5906 × 10^−3^	3.9356 × 10^−3^	2.028 × 10^−6^	6.9848 × 10^−3^	4.8783 × 10^−6^	3.3077 × 10^−8^
F22	Mean	−10.399	−10.396	−10.403	−10.397	−10.403	−10.403
	std	3.8217 × 10^−3^	1.5631 × 10^−2^	2.7891 × 10^−6^	7.5756 × 10^−2^	3.4611 × 10^−6^	5.6416 × 10^−7^
F23	Mean	−10.531	−10.533	−10.536	−10.527	−10.536	−10.536
	std	6.874 × 10^−3^	4.7865 × 10^−3^	3.0245 × 10^−6^	2.1245 × 10^−2^	5.6398 × 10^−6^	6.0013 × 10^−7^
+/=/−		−	1/18 /4	2/8/13	1/18/4	0/4/19	0/4/19
ARV		4.5652	4.217	3.5869	4.217	2.2826	2.0870
Rank		6	4.5	3	4.5	2	1

**Table 8:** Results of MSBWO and the original metaheuristic algorithms.

Fun	Metric	MSBWO	BWO	DBO	GWO	WOA	PSO
F1	Mean	0	1.4907 × 10^−260^	1.4828 × 10^−101^	1.7561 × 10^−33^	3.6949 × 10^−85^	0.1004
	std	0	0	8.1219 × 10^−101^	2.09 × 10^−33^	1.9188 × 10^−84^	0.1005
F2	Mean	1.2231 × 10^−259^	1.4586 × 10^−131^	7.398 × 10^−64^	7.085 × 10^−20^	2.1589 × 10^−51^	4.115 × 10^−2^
	std	0	6.8565 × 10^−131^	4.0335 × 10^−63^	5.5593 × 10^−20^	1.1804 × 10^−50^	2.1195 × 10^−2^
F3	Mean	0	1.7217 × 10^−244^	1.0467 × 10^−60^	5.4544 × 10^−8^	2.5192 × 10^4^	1.1183 × 10^3^
	std	0	0	5.52 × 10^−60^	1.3807 × 10^−7^	1.0986 × 10^4^	9.3686 × 10^2^
F4	Mean	2.7801 × 10^−255^	1.3531 × 10^−128^	3.0078 × 10^−60^	2.3538 × 10^−8^	32.703	5.8991
	std	0	5.1694 × 10^−128^	1.1732 × 10^−59^	2.1745 × 10^−8^	26.172	1.3136
F5	Mean	9.3369 × 10^−8^	2.0861 × 10^−7^	25.082	26.801	27.326	2.5137 × 10^2^
	std	3.6746 × 10^−7^	5.1346 × 10^−7^	0.2056	0.6873	0.3687	5.4071 × 10^2^
F6	Mean	1.7025 × 10^−18^	1.143 × 10^−14^	4.349 × 10^−8^	0.5689	8.4008 × 10^−2^	8.5094 × 10^−2^
	std	3.1276 × 10^−18^	1.437 × 10^−14^	9.5797 × 10^−8^	0.3461	7.317 × 10^−2^	9.0455 × 10^−2^
F7	Mean	4.1334 × 10^−5^	7.2328 × 10^−5^	1.7576 × 10^−3^	1.2252 × 10^−3^	2.938 × 10^−3^	3.2197 × 10^−2^
	std	3.7571 × 10^−5^	5.8612 × 10^−5^	1.1258 × 10^−3^	6.0667 × 10^−4^	2.5824 × 10^−3^	1.1147 × 10^−2^
F8	Mean	−5.559 × 10^112^	−1.2569 × 10^4^	−8.9143 × 10^3^	−6.2936 × 10^3^	−1.1169 × 10^4^	−8.0967 × 10^3^
	std	2.7117 × 10^113^	2.6663 × 10^−9^	1.4104 × 10^3^	4.6811 × 10^2^	1.7026 × 10^3^	6.7177 × 10^2^
F9	Mean	0	0	2.9050	1.2917	1.8948 × 10^−15^	45.336
	std	0	0	7.4451	2.6827	1.0378 × 10^−14^	13.798
F10	Mean	4.4409 × 10^−16^	4.4409 × 10^−16^	4.4409 × 10^−16^	4.2721 × 10^−14^	3.9968 × 10^−15^	0.22272
	std	0	0	0	5.1399 × 10^−15^	2.4685 × 10^−15^	0.3444
F11	Mean	0	0	2.1263 × 10^−3^	2.2938 × 10^−3^	2.7297 × 10^−3^	0.1618
	std	0	0	1.1646 × 10^−2^	6.4787 × 10^−3^	1.4951 × 10^−2^	0.1025
F12	Mean	3.1128 × 10^−11^	1.8546 × 10^−14^	6.1145 × 10^−10^	3.258 × 10^−2^	1.3093 × 10^−2^	2.6513 × 10^−2^
	std	9.8555 × 10^−11^	3.5716 × 10^−14^	1.1332 × 10^−9^	1.7838 × 10^−2^	2.5505 × 10^−2^	5.8293 × 10^−2^
F13	Mean	3.2653 × 10^−11^	1.0811 × 10^−13^	8.5229 × 10^−2^	0.4421	0.2216	0.1118
	std	9.4472 × 10^−11^	1.418 × 10^−13^	0.1234	0.1918	0.1817	0.1284
F14	Mean	0.998	0.998	0.998	2.1503	1.6873	0.998
	std	3.7695 × 10^−12^	6.1182 × 10^−7^	1.3675 × 10^−16^	1.8915	1.8734	4.1233 × 10^−17^
F15	Mean	3.1194 × 10^−4^	3.4182 × 10^−4^	7.5692 × 10^−4^	4.4166 × 10^−3^	7.5181 × 10^−4^	1.7582 × 10^−3^
	std	1.0641 × 10^−5^	4.3411 × 10^−5^	3.7425 × 10^−4^	8.1143 × 10^−3^	3.7044 × 10^−4^	5.0652 × 10^−3^
F16	Mean	−1.0316	−1.0316	−1.0316	−1.0316	−1.0316	−1.0316
	std	1.7061 × 10^−12^	1.3142 × 10^−4^	6.5843 × 10^−16^	1.2002 × 10^−8^	7.3904 × 10^−10^	6.2532 × 10^−16^
F17	Mean	0.3979	0.3992	0.3979	0.3979	0.3979	0.3979
	std	1.1133 × 10^−8^	1.5727 × 10^−3^	0	2.4381 × 10^−5^	3.4691 × 10^−6^	0
F18	Mean	3	3.2594	3	3	3	3
	std	1.0092 × 10^−11^	0.2336	1.5494 × 10^−15^	1.383 × 10^−5^	1.0270 × 10^−5^	1.6223 × 10^−15^
F19	Mean	−3.8628	−3.8594	−3.8622	−3.8616	−3.8604	−3.8628
	std	3.7199 × 10^−5^	2.3940 × 10^−3^	1.9973 × 10^−3^	2.4837 × 10^−3^	3.0790 × 10^−3^	2.6543 × 10^−15^
F20	Mean	−3.3215	−3.2808	−3.2734	−3.2777	−3.2301	−3.2643
	std	2.6160 × 10^−4^	4.2965 × 10^−2^	6.6944 × 10^−2^	7.2579 × 10^−2^	0.2017	6.3786 × 10^−2^
F21	Mean	−10.153	−10.147	−6.2746	−9.1399	−8.9500	−5.8955
	std	3.2037 × 10^−8^	1.1124 × 10^−2^	2.4572	2.0585	2.4501	3.4184
F22	Mean	−10.403	−10.399	−7.6177	−10.401	−9.2588	−7.3590
	std	3.2256 × 10^−7^	5.6792 × 10^−3^	2.8714	8.9801 × 10^−4^	2.3512	3.6012
F23	Mean	−10.536	−10.529	−8.6163	−10.535	−6.9987	−6.9640
	std	8.709 × 10^−7^	1.4087 × 10^−2^	2.7983	6.5498 × 10^−4^	3.6403	3.8923
+/=/−		−	18/3/2	15/7/1	22/1/0	20/3/0	14/7/2
ARV		1.4565	2.7391	3.2609	4.4783	4.3696	4.6957
Rank		1	2	3	5	4	6

**Table 9:** *p* values of the Wilcoxon rank-sum test comparing MSBWO with conventional algorithms on all functions.

Function	BWO	DBO	GWO	WOA	PSO
F1	1.21178 × 10^−12^	1.21178 × 10^−12^	1.21178 × 10^−12^	1.21178 × 10^−12^	1.21178 × 10^−12^
F2	3.01986 × 10^−11^	3.01986 × 10^−11^	3.01986 × 10^−11^	3.01986 × 10^−11^	3.01986 × 10^−11^
F3	1.21178 × 10^−12^	1.21178 × 10^−12^	1.21178 × 10^−12^	1.21178 × 10^−12^	1.21178 × 10^−12^
F4	3.01986 × 10^−11^	3.01986 × 10^−11^	3.01986 × 10^−11^	3.01986 × 10^−11^	3.01986 × 10^−11^
F5	1.44233 × 10^−3^	3.01986 × 10^−11^	3.01986 × 10^−11^	3.01986 × 10^−11^	3.01986 × 10^−11^
F6	3.01986 × 10^−11^	3.01986 × 10^−11^	3.01986 × 10^−11^	3.01986 × 10^−11^	3.01986 × 10^−11^
F7	3.6439 × 10^−2^	3.01986 × 10^−11^	3.01986 × 10^−11^	3.01986 × 10^−11^	3.01986 × 10^−11^
F8	3.01986 × 10^−11^	3.01986 × 10^−11^	3.01986 × 10^−11^	3.01986 × 10^−11^	3.01986 × 10^−11^
F9	1	2.15772 × 10^−2^	3.54361 × 10^−12^	0.33371	1.21178 × 10^−12^
F10	1	1	8.9938 × 10^−13^	3.62921 × 10^−9^	1.21178 × 10^−12^
F11	1	0.33371	4.19262 × 10^−2^	0.33371	1.21178 × 10^−12^
F12	1.42984 × 10^−5^	5.46175 × 10^−9^	3.01986 × 10^−11^	3.01986 × 10^−11^	3.01986 × 10^−11^
F13	6.20265 × 10^−4^	3.01986 × 10^−11^	3.01986 × 10^−11^	3.01986 × 10^−11^	3.01986 × 10^−11^
F14	2.93241 × 10^−10^	1.87212 × 10^−9^	3.05742 × 10^−11^	6.15792 × 10^−11^	1.00916 × 10^−11^
F15	1.06657 × 10^−7^	8.82569 × 10^−7^	0.55923	5.57265 × 10^−10^	3.14633 × 10^−2^
F16	3.00852 × 10^−11^	3.13637 × 10^−12^	3.00852 × 10^−11^	3.24821 × 10^−7^	8.8305 × 10^−12^
F17	3.01986 × 10^−11^	1.21178 × 10^−12^	3.33839 × 10^−11^	2.2539 × 10^−4^	1.21178 × 10^−12^
F18	3.01986 × 10^−11^	2.20334 × 10^−11^	3.01986 × 10^−11^	3.01986 × 10^−11^	2.78095 × 10^−11^
F19	3.01986 × 10^−11^	3.42449 × 10^−8^	1.56381 × 10^−2^	5.09117 × 10^−6^	4.08059 × 10^−12^
F20	3.01986 × 10^−11^	7.24419 × 10^−2^	7.959 × 10^−3^	1.07626 × 10^−2^	0.6586
F21	3.01986 × 10^−11^	1.92277 × 10^−3^	3.01986 × 10^−11^	3.01986 × 10^−11^	5.13599 × 10^−2^
F22	3.01986 × 10^−11^	0.98226	3.01986 × 10^−11^	3.01986 × 10^−11^	0.66015
F23	3.01986 × 10^−11^	2.65493 × 10^−2^	3.01986 × 10^−11^	3.01986 × 10^−11^	0.8762

**Table 10:** Results of MSBWO and five selected SOTA algorithms on 23 benchmark problems.

Fun	Metric	MSBWO	HLOA	HO	PO	CPO	BKA
F1	Mean	0	2.3456 × 10^−240^	0	1.7748 × 10^−47^	2.1585 × 10^−39^	1.237 × 10^−88^
	std	0	0	0	6.7514 × 10^−47^	1.1822 × 10^−38^	6.7752 × 10^−88^
F2	Mean	1.0224 × 10^−259^	1.5503 × 10^−125^	4.7132 × 10^−192^	1.346 × 10^−17^	5.3952 × 10^−22^	3.0805 × 10^−51^
	std	0	5.7134 × 10^−125^	0	7.3722 × 10^−17^	1.9341 × 10^−21^	1.499 × 10^−50^
F3	Mean	0	6.5747 × 10^−237^	0	1.4656 × 10^−35^	1.8336 × 10^−39^	2.9127 × 10^−87^
	std	0	0	0	8.0271 × 10^−35^	9.0774 × 10^−39^	1.5709 × 10^−86^
F4	Mean	1.8495 × 10^−256^	2.4166 × 10^−128^	5.8439 × 10^−191^	1.3277 × 10^−31^	1.0666 × 10^−20^	2.8368 × 10^−44^
	std	0	6.1005 × 10^−128^	0	7.1884 × 10^−31^	4.7757 × 10^−20^	1.2548 × 10^−43^
F5	Mean	5.306 × 10^−8^	24.853	2.4008 × 10^−2^	1.2637 × 10^−3^	25.422	27.303
	std	1.2493 × 10^−7^	9.9102	3.4737 × 10^−2^	2.0162 × 10^−3^	0.2892	1.0939
F6	Mean	6.7936 × 10^−18^	1.5435 × 10^−4^	6.1904 × 10^−3^	1.2590 × 10^−5^	6.6420 × 10^−6^	1.0359
	std	1.9820 × 10^−17^	2.1774 × 10^−4^	8.1137 × 10^−3^	1.7856 × 10^−5^	3.2049 × 10^−6^	0.836
F7	Mean	5.2429 × 10^−5^	2.2378 × 10^−4^	7.8394 × 10^−5^	9.2437 × 10^−6^	1.5765 × 10^−3^	2.1842 × 10^−4^
	std	4.1660 × 10^−5^	2.6434 × 10^−4^	6.9779 × 10^−5^	8.0807 × 10^−6^	9.3270 × 10^−4^	1.8647 × 10^−4^
F8	Mean	−4.7434 × 10^114^	−7.4814 × 10^3^	−2.166 × 10^4^	−6.9567 × 10^3^	−9.0556 × 10^3^	−9.1644 × 10^3^
	std	2.4502 × 10^115^	6.0516 × 10^2^	3.4142 × 10^3^	1.0963 × 10^3^	2.8966 × 10^2^	1.2571 × 10^3^
F9	Mean	0	0	0	0	0	0
	std	0	0	0	0	0	0
F10	Mean	4.4409 × 10^−16^	4.4409 × 10^−16^	4.4409 × 10^−16^	4.4409 × 10^−16^	5.6251 × 10^−16^	4.4409 × 10^−16^
	std	0	0	0	0	6.4863 × 10^−16^	0
F11	Mean	0	0	0	0	0	0
	std	0	0	0	0	0	0
F12	Mean	2.0917 × 10^−11^	1.0387 × 10^−2^	1.4667 × 10^−4^	1.5031 × 10^−6^	1.9325 × 10^−7^	5.4138 × 10^−2^
	std	9.8839 × 10^−11^	3.1659 × 10^−2^	2.9938 × 10^−4^	2.6090 × 10^−6^	1.0923 × 10^−7^	6.4073 × 10^−2^
F13	Mean	3.3187 × 10^−11^	1.9673 × 10^−2^	1.0453 × 10^−3^	5.2258 × 10^−6^	4.0682 × 10^−6^	1.6659
	std	1.0552 × 10^−10^	5.7383 × 10^−2^	2.6180 × 10^−3^	7.9707 × 10^−6^	2.0333 × 10^−6^	0.5876
F14	Mean	1.0975	5.0467	0.998	3.7956	0.998	0.998
	std	0.3995	4.1872	6.2508 × 10^−14^	4.5799	0	1.4283 × 10^−16^
F15	Mean	3.1449 × 10^−4^	7.2813 × 10^−3^	3.075 × 10^−4^	3.0881 × 10^−4^	3.0750 × 10^−4^	1.1101 × 10^−3^
	std	1.6827 × 10^−5^	1.3678 × 10^−2^	1.7418 × 10^−8^	2.0477 × 10^−6^	1.3987 × 10^−8^	4.0566 × 10^−3^
F16	Mean	−1.0316	−1.0316	−1.0316	−1.0316	−1.0316	−1.0316
	std	1.7954 × 10^−11^	5.2156 × 10^−16^	3.4218 × 10^−11^	1.0717 × 10^−10^	6.4539 × 10^−16^	6.1158 × 10^−16^
F17	Mean	0.3979	0.3979	0.3979	0.3979	0.3979	0.3979
	std	2.1712 × 10^−8^	0	2.1185 × 10^−10^	5.6491 × 10^−10^	1.2421 × 10^−13^	0
F18	Mean	3	3	3	3	3	3
	std	6.5647 × 10^−11^	1.4817 × 10^−14^	1.5442 × 10^−9^	1.0930 × 10^−9^	1.3424 × 10^−15^	1.3374 × 10^−15^
F19	Mean	−3.8627	−3.8625	−3.8627	−3.8627	−3.8627	−3.8627
	std	4.7886 × 10^−5^	1.439 × 10^−3^	4.986 × 10^−9^	1.3162 × 10^−5^	2.7101 × 10^−15^	2.5243 × 10^−15^
F20	Mean	−3.3215	−3.2369	−3.2680	−3.2657	−3.3220	−3.3060
	std	2.5687 × 10^−5^	7.5536 × 10^−2^	6.2937 × 10^−2^	7.7621 × 10^−2^	2.8448 × 10^−14^	4.1454 × 10^−2^
F21	Mean	−10.153	−10.149	−10.153	−7.0944	−10.153	−9.6517
	std	3.9089 × 10^−8^	5.8287 × 10^−3^	4.5053 × 10^−7^	2.5402	5.6943 × 10^−15^	1.9085
F22	Mean	−10.403	−9.3208	−10.403	−7.7453	−10.403	−10.403
	std	3.8351 × 10^−7^	2.8136	8.0294 × 10^−7^	2.7031	4.6649 × 10^−16^	9.4054 × 10^−14^
F23	Mean	−10.536	−7.6298	−10.536	−6.3903	−10.536	−10.266
	std	6.9415 × 10^−7^	3.901	5.3435 × 10^−7^	2.3264	2.6182 × 10^−15^	1.4815
+/=/−			16/7/0	8/12/3	13/8/2	12/7/4	15/6/2
ARV		2.2174	4.2861	3	4.1522	3.413	3.9348
Rank		1	6	2	5	3	4

**Table 11:** *p* values of the Wilcoxon rank-sum test comparing MSBWO with selected SOTA algorithms on all functions.

Function	HLOA	HO	PO	CPO	BKA
F1	1.21178 × 10^−12^	1	1.21178 × 10^−12^	1.21178 × 10^−12^	1.21178 × 10^−12^
F2	3.01986 × 10^−11^	3.01986 × 10^−11^	3.01986 × 10^−11^	3.01986 × 10^−11^	3.01986 × 10^−11^
F3	1.21178 × 10^−12^	1	1.65725 × 10^−11^	1.21178 × 10^−12^	1.21178 × 10^−12^
F4	3.01986 × 10^−11^	3.01986 × 10^−11^	3.01986 × 10^−11^	3.01986 × 10^−11^	3.01986 × 10^−11^
F5	3.01986 × 10^−11^	3.01986 × 10^−11^	3.01986 × 10^−11^	3.01986 × 10^−11^	3.01986 × 10^−11^
F6	3.01986 × 10^−11^	3.01986 × 10^−11^	3.01986 × 10^−11^	3.01986 × 10^−11^	3.01986 × 10^−11^
F7	8.12 × 10^−4^	0.13732	2.37682 × 10^−7^	3.01986 × 10^−11^	1.38525 × 10^−6^
F8	3.01986 × 10^−11^	3.01986 × 10^−11^	3.01986 × 10^−11^	3.01986 × 10^−11^	3.01986 × 10^−11^
F9	1	1	1	1	1
F10	1	1	1	0.33371	1
F11	1	1	1	1	1
F12	3.01986 × 10^−11^	3.01986 × 10^−11^	6.72195 × 10^−10^	3.01986 × 10^−11^	3.01986 × 10^−11^
F13	3.01986 × 10^−11^	3.01986 × 10^−11^	1.77691 × 10^−10^	3.01986 × 10^−11^	3.01986 × 10^−11^
F14	1.55785 × 10^−4^	4.8715 × 10^−4^	4.80348 × 10^−6^	3.32524 × 10^−12^	6.17693 × 10^−9^
F15	0.12964	3.01986 × 10^−11^	7.4827 × 10^−2^	3.01986 × 10^−11^	1.06441 × 10^−7^
F16	6.11988 × 10^−11^	3.36405 × 10^−4^	1.55665 × 10^−8^	8.57636 × 10^−12^	2.95423 × 10^−11^
F17	1.21178 × 10^−12^	3.80385 × 10^−7^	2.15403 × 10^−6^	1.72025 × 10^−12^	1.21178 × 10^−12^
F18	4.94171 × 10^−11^	6.73621 × 10^−6^	3.64589 × 10^−8^	5.21145 × 10^−12^	2.56756 × 10^−11^
F19	2.2771 × 10^−10^	3.01986 × 10^−11^	9.46827 × 10^−3^	1.21178 × 10^−12^	1.40589 × 10^−11^
F20	2.27802 × 10^−5^	0.37904	7.7272 × 10^−2^	7.82349 × 10^−12^	1.10772 × 10^−6^
F21	8.48477 × 10^−9^	1.84999 × 10^−8^	2.37147 × 10^−10^	4.08059 × 10^−12^	9.576 × 10^−9^
F22	3.82489 × 10^−9^	0.25805	4.1825 × 10^−9^	2.36567 × 10^−12^	1.69332 × 10^−11^
F23	8.4687 × 10^−9^	0.34783	3.68973 × 10^−11^	3.15782 × 10^−12^	4.24791 × 10^−10^

**Table 12:** Descriptions of datasets.

Symbol	Dataset	No. of Features	No. of Instances
S1	Pima	8	768
S2	Vowel	10	528
S3	Australian	14	690
S4	Zoo	16	101
S5	Vehicle	18	846
S6	Robot	24	5456
S7	Wdbc	30	569
S8	Sonar	60	208
S9	Air	64	359
S10	DNA	180	1186

**Table 13:** Comparison of the BMSBWO with other FS techniques in terms of fitness.

Dataset	BMSBWO	BGWO	BWOA	BDBO	BBWO
S1	0.21587	0.21587	0.21587	0.21587	0.21587
S2	0.13722	0.13722	0.14288	0.14288	0.13722
S3	0.14782	0.15647	0.14782	0.14782	0.14782
S4	0.04117	0.04117	0.04705	0.04117	0.04117
S5	0.24151	0.25288	0.25491	0.24407	0.25825
S6	0.03154	0.03924	0.03789	0.03169	0.03295
S7	0.84448	0.85331	0.85093	0.85127	0.85297
S8	0.09484	0.11405	0.10093	0.12177	0.11077
S9	0.06653	0.07551	0.08910	0.07333	0.07089
S10	0.15851	0.17827	0.17653	0.16958	0.16263
ARV	1.65	3.65	3.65	3.10	2.95
Rank	1	4.5	4.5	3	2

**Table 14:** Comparison of the BMSBWO with other FS techniques in terms of the error rate.

Dataset	BMSBWO	BGWO	BWOA	BDBO	BBWO
S1	0.19047	0.19307	0.19047	0.19047	0.19047
S2	0.08176	0.09182	0.09685	0.09182	0.08301
S3	0.13327	0.14231	0.14711	0.13365	0.13750
S4	0	0	0.02150	0.01075	0
S5	0.23307	0.23937	0.24330	0.23779	0.24094
S6	0.01209	0.01295	0.01661	0.01307	0.01295
S7	0.01099	0.01893	0.01242	0.01832	0.01445
S8	0.07407	0.08994	0.07407	0.10582	0.08994
S9	0.03086	0.04938	0.05864	0.04629	0.04938
S10	0.14606	0.16179	0.16966	0.16067	0.16011
ARV	1.3	3.6	4.1	3.2	2.8
Rank	1	4	5	3	2

**Table 15:** Comparison of the BMSBWO with other FS techniques in terms of the mean feature selection size.

Dataset	BMSBWO	BGWO	BWOA	BDBO	BBWO
S1	0.44444	0.44444	0.44444	0.44444	0.44444
S2	0.6	0.63636	0.63636	0.63636	0.61818
S3	0.29333	0.33333	0.36	0.36	0.33333
S4	0.33333	0.44019	0.44019	0.45098	0.35294
S5	0.41052	0.43157	0.42105	0.42105	0.43157
S6	0.248	0.304	0.248	0.28	0.248
S7	0.18279	0.24731	0.23655	0.27956	0.22580
S8	0.35519	0.37704	0.38797	0.37158	0.36612
S9	0.34871	0.34871	0.39487	0.34871	0.46153
S10	0.35138	0.40883	0.38895	0.40552	0.4022
ARV	1.4	3.75	3.35	3.7	2.8
Rank	1	5	3	4	2

**Table 16:** Comparison of BMSBWO with other FS techniques in terms of average running time.

Dataset	BMSBWO	BGWO	BWOA	BDBO	BBWO
S1	28.45	13.26	13.4	13.42	14.91
S2	27.47	13.11	13.08	13.19	14.34
S3	29.72	13.74	14.01	13.86	15.48
S4	15.64	7.44	7.42	7.41	8.06
S5	43.05	19.88	19.91	20.08	22.35
S6	208.28	97.26	99.69	97.60	108.29
S7	69.74	32.86	33.13	32.65	36.48
S8	25.33	11.74	11.70	11.76	13.27
S9	36.26	16.73	16.65	16.70	18.94
S10	261.69	113.82	117.65	116.65	137.54
ARV	5	1.7	2.1	2.2	4
Rank	5	1	2	3	4

## Data Availability

Data are contained within the article.
